# Emergence of Intergenogroup Reassortant G9P[4] Strains Following Rotavirus Vaccine Introduction in Ghana

**DOI:** 10.3390/v15122453

**Published:** 2023-12-18

**Authors:** Yen Hai Doan, Francis Ekow Dennis, Nobuhiro Takemae, Kei Haga, Hiroyuki Shimizu, Michael Gyasi Appiah, Belinda Larteley Lartey, Susan Afua Damanka, Takaya Hayashi, Toshihiko Suzuki, Tsutomu Kageyama, George Enyimah Armah, Kazuhiko Katayama

**Affiliations:** 1 Center for Emergency Preparedness and Response, National Institute of Infectious Diseases, Tokyo 208-0011, Japan; yendoan@niid.go.jp (Y.H.D.);; 2 Department of Electron Microscopy and Histopathology, Noguchi Memorial Institute for Medical Research, University of Ghana, Accra P.O. Box LG 581, Ghana; 3 Laboratory of Viral Infection, Department of Infection Control and Immunology, Ōmura Satoshi Memorial Institute & Graduate School of Infection Control Sciences, Kitasato University, Tokyo 108-8641, Japan; 4 Department of Virology II, National Institute of Infectious Diseases, Tokyo 208-0011, Japan; 5 Department of Pharmacological Sciences, Icahn School of Medicine at Mount Sinai, New York, NY 10029, USA; 6 Department of Molecular Virology, Graduate School of Medical and Dental Sciences, Tokyo Medical and Dental University, Tokyo 113-8549, Japan; 7 Department of Bacterial Pathogenesis, Infection and Host Response, Graduate School of Medical and Dental Sciences, Tokyo Medical and Dental University, Tokyo 113-8549, Japan

**Keywords:** rotavirus A, G9P[4] strain, DS-1-like, evolution, reassortment, next generation sequencing

## Abstract

Rotavirus (RVA) is a leading cause of childhood gastroenteritis. RVA vaccines have reduced the global disease burden; however, the emergence of intergenogroup reassortant strains is a growing concern. During surveillance in Ghana, we observed the emergence of G9P[4] RVA strains in the fourth year after RVA vaccine introduction. To investigate whether Ghanaian G9P[4] strains also exhibited the DS-1-like backbone, as seen in reassortant G1/G3/G8/G9 strains found in other countries in recent years, this study determined the whole genome sequences of fifteen G9P[4] and two G2P[4] RVA strains detected during 2015–2016. The results reveal that the Ghanaian G9P[4] strains exhibited a double-reassortant genotype, with G9-VP7 and E6-NSP4 genes on a DS-1-like backbone (G9-P[4]-I2-R2-C2-M2-A2-N2-T2-E6-H2). Although they shared a common ancestor with G9P[4] DS-1-like strains from other countries, further intra-reassortment events were observed among the original G9P[4] and co-circulating strains in Ghana. In the post-vaccine era, there were significant changes in the distribution of RVA genotype constellations, with unique strains emerging, indicating an impact beyond natural cyclical fluctuations. However, reassortant strains may exhibit instability and have a limited duration of appearance. Current vaccines have shown efficacy against DS-1-like strains; however, ongoing surveillance in fully vaccinated children is crucial for addressing concerns about long-term effectiveness.

## 1. Introduction

Rotavirus A (RVA) is the predominant etiological agent of acute gastroenteritis in young children worldwide. Each year, it causes more than 110 million diarrheal episodes, 25 million physician visits, 2 million hospitalizations, and 215,000 deaths [1,2]. Although the proportion of RVA detected in children is the same in developed and developing countries, the recorded mortality is much greater in Africa and Asia [2,3].

RVA belongs to the *Reoviridae* family. Its genome comprises 11 segments of double-stranded RNA that encode six structural viral proteins (VPs) and six nonstructural proteins (NSPs) [4]. RVAs have been classified using various approaches. Specifically, they have been categorized based on the antigenic properties of VP6, VP7, and VP4 (sub-groups and serotypes); the migration pattern of the RNA genome segments when subjected to polyacrylamide gel electrophoresis (long, short, and super short electropherotypes); the whole-genome RNA hybridization patterns; and the nucleotide sequence analysis (genotypes). The G and P serotypes are defined by the antigenicity of the outer capsid neutralization proteins, VP7 and VP4, respectively. These serotypes are often referred to as G and P genotypes, respectively, because molecular assays are more commonly used for their determination than are serologic assays. The genotype classification has been expanded to include all 11 genome segments. This classification system denotes the VP7-VP4-VP6-VP1-VP2-VP3-NSP1-NSP2-NSP3-NSP4-NSP5/6 genes of an RVA strain as a descriptor Gx-P[x]-Ix-Rx-Cx-Mx-Ax-Nx-Tx-Ex-Hx (x indicating genotype number), respectively. With the application of the nucleotide sequence-based genotype classification system, the Wa, DS-1, and AU-1 genogroups, which were redefined as genotype constellations, are described as G1/G3/G4/G9-P[8]-I1-R1-C1-M1-A1-N1-T1-E1-H1, G2/G8-P[4]-I2-R2-C2-M2-A2-N2-T2-E2-H2, and G3-P[9]-I3-R3-C3-M3-A3-N3-T3-E3-H3, respectively [5]. 

The research suggests that RVA vaccines are most effective at preventing the most severe and life-threatening cases of RVA. The first widely used RVA vaccine was approved in the United States in 2006. Today, there are four oral RVA vaccines recommended for use by the World Health Organization (WHO): Rotarix^®^ (GlaxoSmithKline Biologicals), RotaTeq^®^ (Merck), RotaSiil^®^ (Serum Institute of India), and Rotavac^®^ (Bharat Biotech) [6]. Among these vaccines, Rotarix^®^ and RotaTeq^®^ are the most widely used, as both have shown good efficacy against RVA infections in clinical trials and real-world settings [7,8]. Since the approval of RVA vaccines, they have had a notable impact on the reduction of RVA-related deaths. According to a study published in 2018, the use of RVA vaccines prevented approximately 28,900 child deaths globally in 2016 [9]. However, the widespread use of RVA vaccines would result in selective evolutionary pressure resulting in strain replacement. Recently, a larger proportion of reassortant DS-1-like strains (G1/G3/G8/G9-P[8]/P[4]/P[6]-I2-R2-C2-M2-A2-N2-T2-E2-H2) were reported in many countries. While unusual, G1/G3/G8 DS-1-like strains have emerged in many parts of the world (Africa, Asia, Australia, Europe, and the Americas) [10,11,12,13,14,15,16,17,18]; G9P[4] DS-1-like strains have only been found with high prevalence in Latin American countries, India, Bangladesh, and Iran [19,20,21,22]. Although, G9P[4] strains have also been reported in the US, Japan, Italy, South Korea, and the Czech Republic, they only appear sporadically in these countries [16,23,24,25,26]. The first appearance of reassortant G9P[4] was reported in Latin American countries after a few years of RVA vaccine introduction, possibly due to the selective pressure. However, this strain also showed a significant increase in prevalence in Bangladesh and India during 2010–2013 when RVA vaccines had not been introduced into the national immunization program. Thus, it remains unclear whether the observed increased detection of reassortant G9P[4] strains is due to RVA vaccine introduction or due to the genetic background fluctuations.

In Ghana, during the pre-vaccine period, the G1, G2, and G3 genotypes of VP7 genes, in conjunction with the P[8] and P[6] genotypes of VP4, were the predominant genotypes, accounting for over 65% of the circulating G and P types. However, in the first three RVA seasons after the introduction of the vaccine (2012–2015), the G12P[8] and G10P[6] genotypes became the most prevalent, representing 36% of the circulating G and P types. Notably, in the fourth year following the introduction of the RVA vaccine, we observed the emergence of the G9P[4] RVA strain [27], which had initially been detected in Ghana in the early 2000s. Its successive detection occurred in 2013 with a prevalence of 2 out of 144 (1.4%), and it spiked in 2016 with a prevalence of 8 out of 51 (15.7%) [27].

To investigate whether Ghanaian G9P[4] strains also exhibited the DS-1-like backbone, as seen in G9P[4] strains detected in India between 2011 and 2013, and the G1/G3/G8 DS-1-like strains found in Asian countries from 2011 to 2016, this study aimed to analyze the whole genome sequences of fifteen G9P[4] and two G2P[4] RVA strains identified in Ghana during 2015–2016. The results of this study can shed light on the origin of G9P[4] in Ghana and may provide evidence that the appearance of these unusual G9P[4] strains was influenced by selective pressures stemming from RVA vaccines.

## 2. Materials and Methods

### 2.1. Specimens and Ethical Approval

A total of 75 stool specimens were collected from children less than five years of age with acute gastroenteritis at the Navrongo War Memorial Hospital in the Upper East region of Ghana between 2015 and 2016. Additionally, during the same period, 583 samples and 126 samples were also collected from children less than five years of age with acute gastroenteritis at hospitals in Accra (Greater Accra region) and Sekondi (Western region), respectively (Table 1). Gastroenteritis was defined as three or more passages of watery diarrhea or looser-than-normal stool within 24 h. This study was conducted in accordance with the ethical clearance and was approved on 20 November 2015 by the Institutional Review Board of the Noguchi Memorial Institute for Medical Research (NMIMR), Ghana (IRB 00001276). All stool samples were initially screened for RVA using enzyme immunoassay (ProSpecT^TM^, Oxoid Cambridge, UK) at the NMIMR, University of Ghana. The G and P genotypes of all RVA-positive specimens were examined by RT-PCR [27].

### 2.2. RNA Extraction

The RVA dsRNA was extracted from 10% stool suspensions by the phenol/chloroform method following the protocol described in a previous study [28] and was purified with an RNaid Kit (Bio 101, Carlsbad, CA, USA).

### 2.3. cDNA Library Preparation and Illumina MiSeq Sequencing

cDNA library preparation and Illumina MiSeq sequencing were conducted following previously established protocols [20]. Briefly, individual strains were subjected to library construction using a NEBNext Ultra RNA library Prep Kit for Illumina v1.2 (New England Biolabs, Ipswich, MA, USA), incorporating bar-coded adapters to generate a 200 bp fragment library. The resulting libraries were purified using Agencourt AMPure XP magnetic beads (Beckman Coulter, Brea, CA, USA) and were assessed for quality on a MultiNA MCE-202 bioanalyzer (Shimadzu Corporation, Kyoto, Japan). Nucleotide sequencing was performed on an Illumina MiSeq sequencer (Illumina, San Francisco, CA, USA) with a MiSeq Reagent Kit v2 (Illumina) to generate 151 paired-end reads. Data analysis was carried out with CLC Genomics Workbench v22 (CLC Bio, Tokyo, Japan). The complete or nearly complete nucleotide sequence of each gene segment of Ghanaian RVA strains was obtained by de novo assembly and mapping reads to reference in CLC Genomics Workbench.

### 2.4. Genetic Analysis Algorithms

Genotyping: Genotypes of confirmed RVA gene segments were determined using the automated genotyping tool Viral Pathogen Database and Analysis Resource [29].

Selecting reference sequences for phylogenetic analyses: To interpret the evolution of Ghanaian G9P[4] RVA strains at the lineage level, we utilized all reference sequences of the G2P[4] and G9P[4] strains from our previous paper [20] and a set of reference strains for genotype 2 genes [30] for phylogenetic tree analyses in this study. Additionally, we collected nucleotide sequences carrying the G9, P[4], I2, R2, C2, M2, A2, N2, T2, and H2 genotypes, detected in Ghana, by applying specific filters on the RVA database of the NCBI Virus Variation Resource. Sequences longer than 90% of the full length of each segment were included in our analysis.

To compile the NSP4 gene sequences for the E6 genotype, we utilized the BLAST program (http://blast.ncbi.nlm.nih.gov/, accessed on 23 October 2023) with the NSP4 sequence of our G9P[4] as the query. To ensure the inclusion of all relevant sequences, we increased the ‘Max target sequences’ option in the BLAST program to 250. A preliminary phylogenetic tree was constructed using the MEGA 6.0 software package [31] to identify the E6 genotype cluster, using outgroup strains as indicators and removing sequences from other genotypes. From this analysis, we obtained 51 nucleotide sequences for the E6 genotype; however, only sequences longer than 90% of the full length of the NSP4 gene were retained for further analysis. Additionally, we included eight NSP4 sequences of the E1, E2, and E3 genotypes, which exhibited the closest similarity to the E6-NSP4 sequences based on BLAST research results, in the NSP4 phylogenetic tree.

Phylogenetic analysis: The nearly full-length genome sequences were aligned with reference sequences using the MAFFT multiple sequence alignment program, version 7.0 [32]. The selection of the best substitution models for phylogenetic tree construction was based on the corrected Akaike Information Criterion (AICc) value, implemented in MEGA6 [31]. The following substitution models were used in this study: Tamura 3-parameter (T92) + G for VP7, VP4, NSP1, NSP3, NSP4, and NSP5; T92 + G + I for VP1, VP6, and NSP2; Tamura-Nei (TN93) + G + I for VP2; and General Time Reversible (GTR) + G + I for VP3. For each genome segment, a maximum likelihood tree was constructed using the MEGA6 package. Lineage designations were defined based on previous studies for VP7-G9, NSP4-E6, and genotype 2 genes [20,30,33].

Proportional p-distances of nucleotide sequences: The genetic distance (p-distance) was calculated using the MEGA6 package. Figures and a statistical analysis of the p-distance were generated using GraphPad Prism version 9.

### 2.5. Nucleotide Sequence Accession Numbers

The nucleotide sequences described in this study were deposited to DDJB/GenBank/EMBL databases under the accession numbers OR890100 to OR890286.

## 3. Results

### 3.1. The Collection of Samples, Age Distribution, and Proportion of Rotavirus Detection

A total of 784 stool specimens were collected from children experiencing acute gastroenteritis in three distinct regions of Ghana between 2015 and 2016. Among these samples, 142 (18%) were obtained from children under 6 months old, 240 (31%) from those aged 6 to 12 months, 284 (36%) from the 13–24-month age group, and 118 (15%) from children older than 24 months. All collected samples underwent screening for RVA using enzyme immunoassay, revealing that 217 (27.7%) samples tested positive for RVA. Upon categorizing the proportion of RVA-positive cases by age group, the detection rate ranged from 17.1% to 25.2% across the four age groups. A further analysis of RVA detection rates in different regions showed the highest rate in the Western region at 50.8%, followed by 36% in Navrongo and 21.6% in Greater Accra (Table 1).

### 3.2. Genotype Constellation

Among the RVA-positive samples, we selected the 15 G9P[4] RVA strains from the 2015–2016 RVA season, taking into consideration the sufficient volume of samples available for subsequent whole-genome characterization. These selected strains were then sent to the National Institute of Infectious Disease in Tokyo, Japan for next-generation sequencing (NGS) analyses. These samples included seven collected from the Greater Accra region (named as 039M, 082M, 103M, 110M, 119M, 132M, and 135M); seven collected from the Navrongo in the Upper East regions (named as WMH-1439, WMH-1441, WMH-1445, WMH-1446, WMH-1451, WMH-1452, and WMH-1453); and one additional sample (named as EUHC-002) collected in the Western region of Ghana. Complete nucleotide sequences for 11 genome segments of these 15 G9P[4] strains were determined by the short reads NGS using the Illumina MiSeq platform. All fifteen Ghanaian G9P[4] strains were double-reassortant strains carrying the G9-VP7 and E6-NSP4 genes on a DS-1-like genetic background (G9-P[4]-I2-R2-C2-M2-A2-N2-T2-E6-H2). Two Ghanaian G2P[4] strains were typical DS-1 strains (G2-P[4]-I2-R2-C2-M2-A2-N2-T2-E2-H2). We conducted a comparison of the genotype constellations of the newly identified Ghanaian G9P[4] strains with other G9P[4] strains available in the GenBank databases. Our analysis revealed that the genotype constellations of the Ghanaian G9P[4] strains closely resemble those of six of the G9P[4] strains that were highly prevalent in Pune and Kolkata, India during 2010–2013 (G9-P[4]-I2-R2-C2-M2-A2-N2-T2-E6-H2). However, the Ghanaian G9P[4] strains differ from sporadic G9P[4] strains detected in Japan, Bangladesh, Paraguay, South Korea, Italy, and the Czech Republic between 2007 and 2018. Notably, only one sporadic G9P[4] strain detected in the USA in 2010 (LB1562) exhibited a similar genotype constellation to the Ghanaian G9P[4] strains identified in our study (Supplementary Table S1). 

### 3.3. Comprehensive Collection of RVA Genotype Constellations in the Pre-Vaccination Era

**Figure 1:**
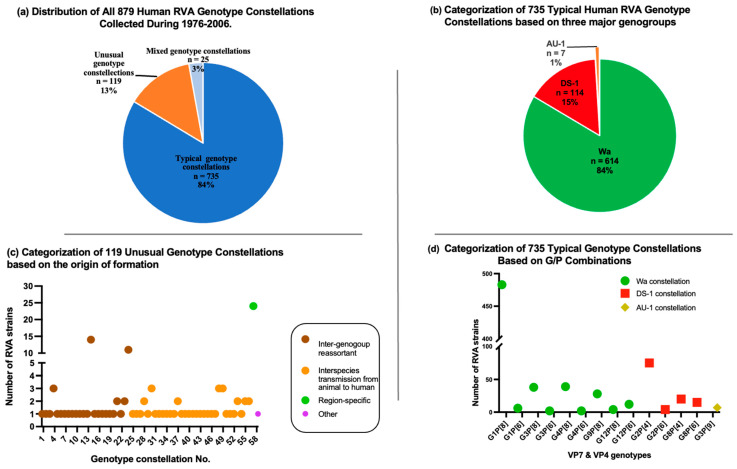
Genotype Constellations of Human RVAs Detected Before Vaccine Introduction (1976–2006). (**a**) Distribution of all 879 human RVA genotype constellations based on three classifications: typical, unusual, and mixed-genotype constellations. (**b**) Distribution of 735 typical human RVA genotype constellations categorized into three major genogroups: Wa, DS-1, and AU-1. (**c**) Distribution of 119 unusual genotype constellations. Within this set of unusual RVA strains, there were 58 distinct genotype constellations (*X*-axis). Various dot colors denote different unusual constellation groups, reflecting their origin of formation: intergenogroup reassortant, interspecies transmission from animal to human, and rare genotypes in specific geographical regions. (**d**) The number of typical human RVA genotype constellations based on their VP7 and VP4 genotypes. Different dot colors represent different genetic backbones: Wa, DS-1, and AU-1.

To assess the changes in the distribution of genotype constellations in recently reported RVA strains and to determine whether these changes are attributed to natural cyclical fluctuations of predominant circulating strains or the impact of the RVA vaccine, we gathered all available data on RVA genotype constellations detected before the introduction of the RVA vaccine (during the period of 1974–2006) from the DNA database. We then compared these findings with those of the RVA strains determined in recent years. A total of 879 RVA genotype constellations from human RVA strains isolated between 1974 and 2006 were collected, representing 27 countries across five continents. Among these 879 RVAs, 735 strains carried the typical human RVA genotype constellations, 119 strains carried unusual genotype constellations, and 25 strains had mixed genotypes (Figure 1a and Supplementary Table S2). 

Out of 735 typical RVA strains, 614 (84%) exhibited the G1, G3, G4, G9, and G12 genotypes in combination with P[8] and P[6], carrying the Wa genotype constellation. Additionally, 114 (15%) strains showcased the G2 and G8 genotypes in combination with P[8] and P[6], featuring the DS-1 genotype constellation. Only 7 (1%) of the strains were identified as the G3P[9]-AU-1 genotype constellation. The most predominant genotype constellation was G1P[8] Wa-like, accounting for 484 strains, followed by G2P[4] DS-1-like with 75 strains (Figure 1b,d and Supplementary Table S2a). 

In our study, unusual RVA genotype constellations were defined based on criteria such as constellations resulting from intergenogroup reassortment strains, human RVAs with at least one genome segment originating from animal RVA strains, or genotypes exclusive to a specific geographical region. Remarkably, only 13% of the RVA strains collected during 1976 and 2006 carried the unusual genotype constellations (Figure 1a and Supplementary Table S2). Among these unusual RVA strains, there were 58 distinct genotype constellations. It is noteworthy that most of these unusual genotype constellations were sporadically detected, except for two unusual genotype constellations that exhibited repeated circulation and were detected with high prevalence in a specific geographical region. The first exception is the mono-reassortant G2P[4] strains that exhibited repeated circulation over a period of 6 years (1985–1990) in Japan [34]. The second exception is the high detection rate of an unusual RVA strain (G10-P[11]-I2-R2-C2-M2-A1-N1-T1-E2-H3) in neonatal nurseries in India (Figure 1b and Supplementary Table S2b). 

Among the 879 RVA strains collected before the introduction of the vaccine, there were 25 cases of mixed infections. Mixed genotypic constellations were identified when at least one genome segment had at least two sequences with either distinct genotypes or identical genotypes. These mixed genotypes occurred between two viruses within the same genogroups, as well as between two viruses from different genogroups (Figure 1a and Supplementary Table S2c).

### 3.4. Phylogenetic Analysis and Proportional p-Distances

The Ghanaian G9P[4] strains shared genotype 2 with DS-1-like RVA strains for the VP4, VP6, VP1–VP3, NSP1–NSP3, and NSP5 genes. Our objective was to determine the similarity between these genes of G9P[4] strains and contemporary DS-1-like RVA strains. To achieve this, we conducted phylogenetic tree analyses using reference sequences of G2P[4] and G9P[4] strains from our previous paper [20], a set of reference strains for genotype 2 genes [30], and nucleotide sequences of G9, P[4], I2, R2, C2, M2, A2, N2, T2, E6, and H2 genotypes detected in Ghana. Based on the recently proposed lineage designation for genotype 2 of globally circulating DS-1-like strains and genotype 2 and 9 for VP7 genes [20,30,33], the Ghanaian G9P[4] strains belonged to major sub-lineage III for VP7; lineage IVa for VP4, VP2, NSP1, and NSP5; lineage V for VP1, VP6, NSP2, and NSP3; and lineage VII for VP3 (Supplementary Figure S3). These lineages were identified as emergent lineages of contemporary human RVA strains in previous studies [30,33]. This study revealed that the G9P[4] strains in Ghana were closely related to contemporary human RVA strains, with no evidence of genome segments derived from animal RVA strains.

**Figure 2:**
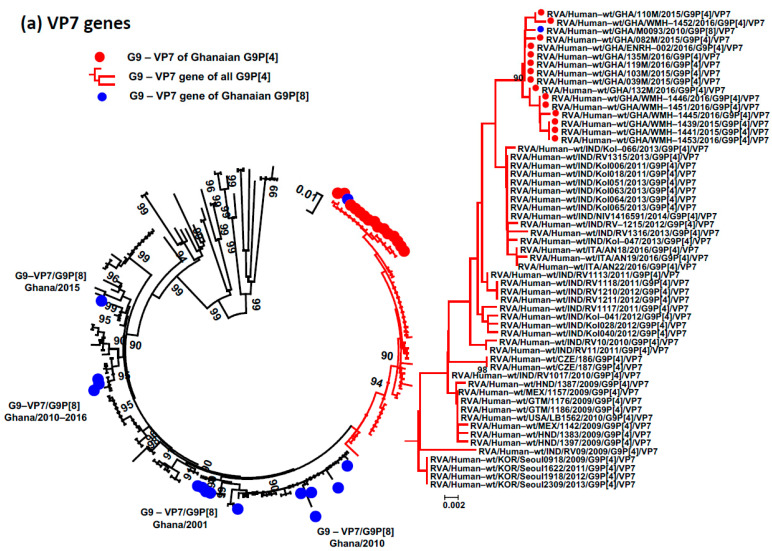

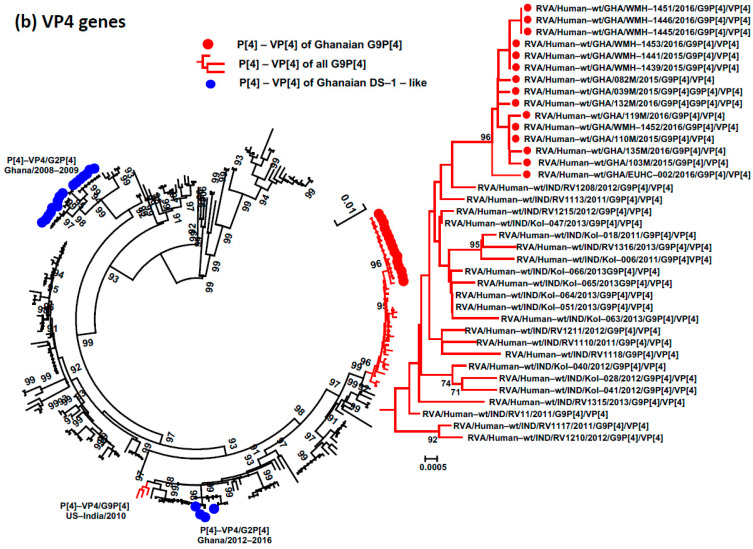

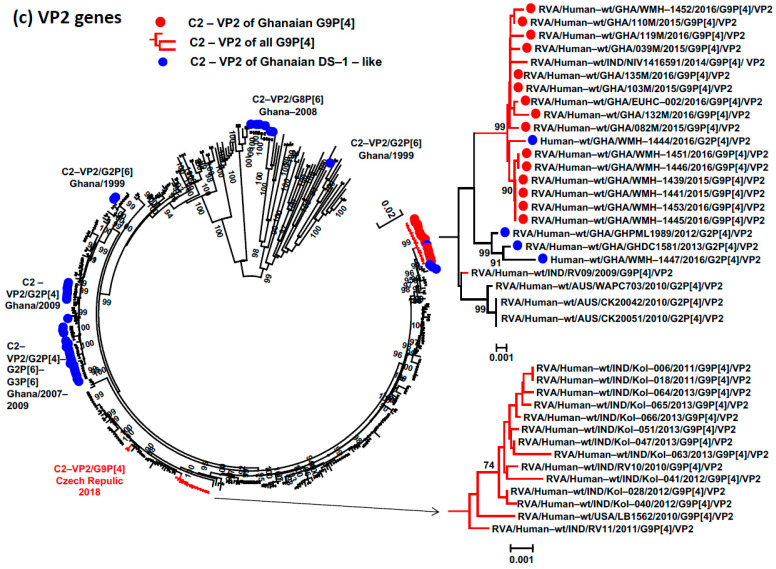

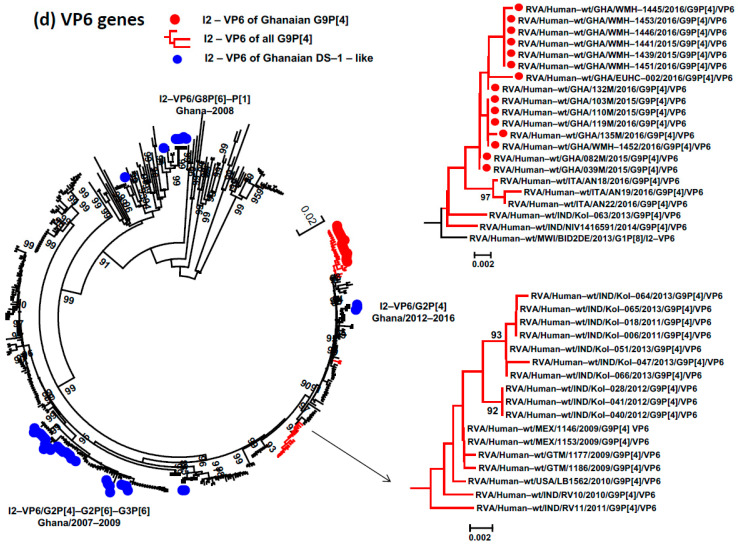
Simplified phylogenetic trees were constructed for 4 genome segments: VP7 (**a**), VP4 (**b**), VP6 (**c**), and VP2 (**d**). The tree includes the 15 Ghanaian G9P[4] strains from this study (indicated by red dots), as well as additional G9P[4] strains (indicated by red branch trees). Furthermore, two Ghanaian G2P[4] strains from this study, along with Ghanaian strains carrying G9/VP7, P[4]/VP4, and genotype 2 for the VP6 and VP2 genome segments, were incorporated into the analysis (indicated by blue dots). Global reference RVAs carrying G9/VP7, P[4]/VP4, and genotype 2 for VP6 and VP2 were also integrated into the trees. The trees were generated using the maximum likelihood method within the MEGA6 software package, with bootstrap values determined from 1000 replicate trials. Genetic distances are indicated at the bottom, and the percent bootstrap support is displayed at each node when it reaches 70% or higher.

**Figure 3:**
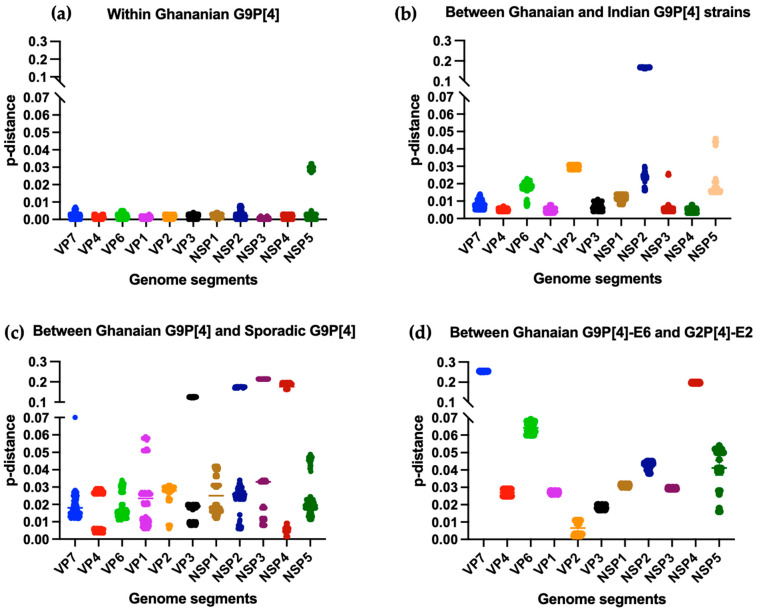
Proportional p-distances of nucleotide sequences between Ghanaian G9P[4] strains and other RVA strains in 11 genome segments (**a**) within 15 Ghanaian G9P[4] strains, (**b**) between Ghanaian G9P[4] and predominant G9P[4] strains in India, (**c**) between Ghanaian G9P[4] and sporadic G9P[4] strains in other countries, and (**d**) between Ghanaian G9P[4] and Ghanaian G2P[4] strains. Color coding of each genome segment is the same as in (**a**–**d**).

**Figure 4:**
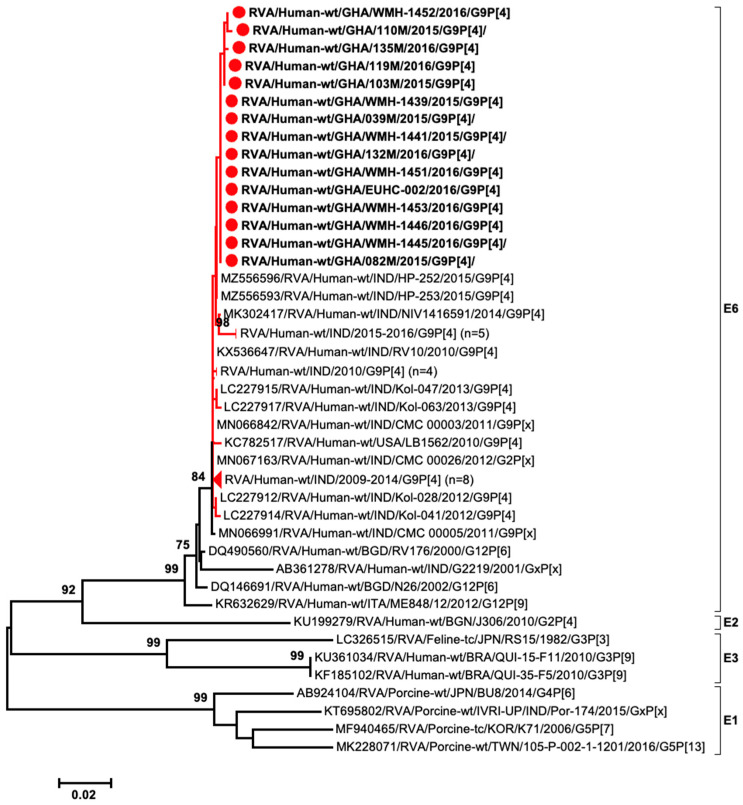
Phylogenetic tree of NSP4 gene nucleotide sequences of 15 Ghanaian G9P[4] strains in this study (indicated by red dots). The tree also includes 28 G9P[4] strains detected in India and the USA (indicated by red branch trees), along with five other RVA strains for which the NSP4 gene was E6 genotype. Additionally, eight NSP4 sequences of the E1, E2, and E3 genotypes, which exhibited the closest similarity to the that of the E6 genotype, are included in the tree. To simplify the tree and enhance visibility, certain sequences were collapsed into read triangles with the number of collapsed sequences is indicated at the end of each line. This tree was constructed using the maximum likelihood method that is included in the MEGA software package (version 6) with bootstrap values after 1000 replicate trials. The genetic distance of this tree is indicated at the bottom. Percent bootstrap support is shown by the value at each node when it is 70% or larger.

The phylogenetic trees showed that the branch topology of most segments of the Ghanaian G9P[4] strains fell within the same monophyletic lineage, indicating a high nucleotide identity (99.2–100%) among these strains. However, the NSP5 genes of WMH-1445, WMH-1453, and EUHC-002 did not cluster with other NSP5-H2 genes of Ghanaian G9P[4] strains, exhibiting a lower nucleotide identity (96.8–97.2%) (Figure 2, Figure 3 and Figure 4; Supplementary Figure S1). To understand the relationship between Ghanaian G9P[4] and other RVA strains, we compared the Ghanaian G9P[4] strains to previously reported endemic G9P[4] strains and other Ghanaian RVA strains that carried at least one of the G9, P[4], I2, R2, C2, M2, A2, N2, T2, E6, and H2 genotypes. The VP7, VP4, VP1, VP3, NSP1, NSP3, and NSP4 genes of Ghanaian G9P[4] strains fell within the same monophyletic lineage as those of Indian G9P[4] strains detected in Kolkata with high prevalence during 2011–2013. However, the VP2, VP6, NSP2, and NSP5 genes of the Ghanaian G9P[4] strains did not cluster with those of the Indian G9P[4] strains detected in Kolkata (Figure 2c,d and Supplementary Figure S1d,f). In these genome segments, the Ghanaian G9P[4] strains clustered with the G2P[4] RVA strains detected in Ghana during 2008 and 2016, indicating intra-reassortment events between the original G9P[4] strains and co-circulating strains in Ghana. It is noted that the VP7 sequence of one Ghanaian G9[8] strain detected in 2010 clustered within the monophyletic lineage of the VP7 genes of all 15 Ghanaian G9P[4] strains, suggesting that the VP7 gene of the G9P[4] strain might have appeared in Ghana since 2010 or earlier (Figure 2a). This study does not provide conclusive evidence regarding the migration of reassortant G9P[4] strains from India to Ghana.

To assess the genetic diversity among G9P[4] strains, we compared the nucleotide sequences of each of the 11 segments of the newly determined Ghanaian G9P[4] strains in this study with those of segments carrying the same G9, P[4], I2, R2, C2, M2, A2, N2, T2, E6, and H2 genotypes of the endemic G9P[4] strains detected in Kolkata, India during 2011–2013. We also compared them to the sequences of sporadic G9P[4] strains. The VP7, VP4, VP1, VP3, NSP1, NSP3, and NSP4 genes of Ghanaian and Kolkata, Indian G9P[4] strains demonstrate a close genetic relatedness, with a mean p-distance ranging from 0.004 to 0.012 (nucleotide identities of 98.8% to 99.6%). However, their VP6, VP2, NSP2, and NSP5 sequences exhibited higher genetic differences, with a mean p-distance ranging from 0.016 to 0.03 (nucleotide identities of 97% to 98.4%) (Figure 3b). The Ghanaian G9P[4] strains also showed lower homology when compared to segments with the same G9, P[4], I2, R2, C2, M2, A2, N2, T2, and H2 genotypes from sporadic G9P[4] strains (97.2% to 99.4%) (Figure 3c). The high degree of genetic similarity of the VP7, VP4, VP1, VP3, NSP1, NSP3, and NSP4 genes among Ghanaian and Indian G9P[4] strains, as demonstrated in this study, suggests that the endemic G9P[4] strains from Ghana and India had a common origin. The lower homology between the VP6, VP2, NSP2, and NSP5 genes of the Ghanaian and Indian G9P[4] strains was likely generated from further intra-reassortment events between the original G9P[4] strains and co-circulating strains in Ghana. The observation of the VP2 gene is one example of intra-reassortment events between the original G9P[4] strains and co-circulating strains in Ghana. While the genetic distance of the VP2 gene between the Ghanaian G9P[4] strains and previously reported G9P[4] strains is significant (mean of p-distance was 0.028), and their VP2 gene exhibits close genetic relatedness to that of the co-circulating G2P[4] strains detected in Ghana from 2012 to 2016 (mean of p-distance was 0.006) (Figure 3d). 

### 3.5. The Circulation of the Uncommon E6 Genotype of the NSP4 Gene

To investigate the origin of the E6-NSP4 genotype in Ghanaian G9P[4] strains, we conducted a comprehensive search in the GenBank database to obtain available E6 sequences. A total of 51 nucleotide sequences for the E6 genotype were found, all of which belonged to RVA strains isolated from humans between 2000 and 2016. In the phylogenetic tree of E6-NSP4, all 15 newly identified Ghanaian E6 sequences formed a distinct cluster along with other E6 sequences from the G9P[4] strains available in the GenBank database. This cluster separated from the E6 sequences of the G12P[6], G12P[9], and G8P[6] strains. The sequences of one human G2P[4]-E2, one feline G3P[3]-E3, two human G3P[9]-E3, and four porcine G4/G5-E1 strains exhibited the highest similarity to the E6 genotype (Figure 4). The genetic similarity between the E6 sequences of G9P[4] strains and the E6 sequences of other strains (G12P[6], G12P[9], and G8P[6]) ranged from 97.6 to 100% (Supplementary Figure S2). 

## 4. Discussion

RVA vaccines have consistently demonstrated excellent safety and efficacy profiles in clinical trials and real-world settings. However, two significant issues still require attention and further investigation. First, there is a possibility that widespread use of the RVA vaccine may exert selective evolutionary pressure, leading to strain replacement. Second, the efficacy of the RVA vaccine is reduced in developing countries, where the burden of RVA-related morbidity and mortality is highest. To address the first concern, numerous countries and researchers worldwide are actively monitoring and analyzing the genotypic characterization and evolution of RVA strains post-vaccination. 

Ghana introduced an RVA vaccine into their national immunization program in 2012. The percentage of hospital admissions positive for RVA before vaccine introduction was around 48% [35]. This study indicates that the RVA detection rate fell to 27.7% after four years of the RVA vaccine introduction (Table 1). However, when the average of the RVA detection rate is broken down by different regions, we observe that the detection rate remains high in the Western region of Ghana with 50.8%, while it has reduced to 36% in Navrongo, Upper East region and 21.6% in Greater Accra (Table 1). Despite uniform RVA vaccine coverage across all regions of the country, further studies need to understand the variations in RVA vaccine efficacy in different regions of Ghana. 

A reduction in the number of children requiring hospitalization due to RVA infection in Ghana was observed in this study and in another report [35]. However, during RVA surveillance in Ghana, we found that the reassortant G9P[4] strain emerged in the fourth year following the introduction of the Rotarix vaccine [27]. Remarkably, this study discovered that this reassortant G9P[4] strain carried the DS-1 genomic backbone, which is consistent with recent reports of the emergence and widespread circulation of intergenogroup reassortant strains that carried the DS-1 backbone in Asia and other countries [10,11,12,13,14,15,16,17,18].

The sporadic detection of reassortant G9P[4] RVAs was reported before the introduction of the RVA vaccine [36,37,38]. The genetic background of Ghanaian G9P[4] strains is different with that of sporadic, detected G9P[4] strains, except for one sporadic G9P[4] strain in the US. And they are only similar with the endemic G9P[4] strains in other countries in only seven genome segments (VP7, VP4, VP1, VP3, NSP1, NSP3, and NSP4), while the remaining four genome segments (VP6, VP2, NSP2, and NSP5) are close to those of the Ghanaian G2P[4] strains detected in 2012–2016. The VP7 sequence of one Ghanaian G9[8] strain detected in 2010 clustered within the monophyletic lineage of the VP7 genes of all 15 Ghanaian G9P[4] strains, suggesting that the VP7 gene of the G9P[4] strain might have appeared in Ghana since 2010 or earlier (Figure 2a). Therefore, it is unclear whether the reassortant G9P[4] strain was introduced from other regions to Ghana. However, the obtained results suggest that Ghanaian G9[4] strains share the same ancestor with the endemic G9P[4] DS-1-like strains detected in India and other countries, and they made further intragenotype reassortants with the co-circulating strains in Ghana.

The Bayesian analysis conducted in our previous study indicated that the reassortant G9P[4] DS-1-like strains appeared before the global use of RVA vaccines [20]. Moreover, the emergence of G9P[4] DS-1-like strains was observed in countries with a low RVA vaccine coverage at the time of study, such as India and Bangladesh [20,22], and in countries with a high RVA vaccine coverage, like Ghana and Latin American countries [21]. These results might indicate that the reassortant G9P[4] DS-1-like strain was generated in the pre-vaccination era. When the RVA vaccine was introduced into the human population, it might have provided an advantage for this reassortant G9P[4] strain to spread and become predominant in Ghana and other countries.

The widespread reproduction and circulation of recombinant strains, such as G9P[4] and other DS-1-like strains, such as G1P[8], G3P[8], and G8P[8], raise the question of whether they are driven by vaccine pressure or natural cyclical fluctuations of predominant circulating strains. To address this question, our study investigated changes in RVA genotype constellations at the genotype level before and after the introduction of rotavirus vaccines. We compiled comprehensive information on the genotype constellations of RVA strains pre- and post-vaccine introduction. Our study showed that only a small portion (13%) of RVA strains detected before 2006 carried unusual genotype constellations. It is worth noting that the majority of these unusual genotype constellations were sporadically detected. However, there was an exceptional case of mono-reassortant G2P[4] strains that demonstrated repeated circulation over a period of 6 years in Japan. Specifically, intergenogroup mono-reassortant G2P[4] RVA strains carrying the N1 genotype of the NSP2 gene were identified in Japan between 1981 and 1991 [34]. Additionally, there was another exception with a high detection rate of an unusual RVA strain (G10-P[11]-I2-R2-C2-M2-A1-N1-T1-E2-H3) in neonatal nurseries in India. Some argue that the recent discovery of many reassortant RVA strains is due to the rapid development of RVA detection techniques, particularly the widespread use of sequencing technology, which facilitates easier genome characterization and consequently results in a higher prevalence of reassortant strains. However, many studies have determined the whole genome of RVAs from archived specimens collected before 2006 [39,40,41,42,43,44]. These results do not indicate that recombinant strains were highly prevalent in RVA strains before the RVA vaccine introduction. In comparison to the prevalence of recombinant strains in the period after vaccine introduction, the distinct change in distribution patterns of divergent strains, reflected in the emergence of RVA genotypes in the post-vaccine era, appears unique and beyond the level of natural cyclical fluctuations. With the widespread emergence of recombinant strains carrying the DS-1 genomic backbone, which is completely different from the Wa-like backbone of the monovalent rotavirus vaccine strain used globally, there is concern about the vaccine’s effectiveness against DS-1-like strains. However, several studies have shown that the RVA vaccine is effective in preventing severe gastroenteritis caused by G9P[4] DS-1-like and other DS-1-like RVA strains. These findings suggest that the existing RVA vaccines offer protection even against these genetically distinct RVA strains [45,46,47,48,49]. 

The uncommon E6 genotype was initially identified in a G8P[6] strain in India in 2000. Although this G8P[6] strain was isolated from humans, it was reported that the G8-VP7 gene originated from bovines [50]. Additionally, the E6 genotype was detected in two G12[P6] DS-1-like strains, namely RV176-00 and N26-02, in Bangladesh in 2000–2001 [51]. Furthermore, a multi-reassortant G12P[9] RVA with novel VP1, VP2, VP3, and NSP2 genotypes carrying the E6 genotype was identified in Italy in 2012 [52]. Moreover, the sequences of the E1 and E3 genotypes that were detected from feline and porcine RVA strains were shown to be the closest sequences of the E6 genotype from the BLAST search. Therefore, the E6 genotypes might have originated from animal RVA strains. It is worth noting that the presence of the E6 genotype in G8 and G12 RVA strains was sporadic. However, its significance became apparent only with the emergence and widespread prevalence of G9P[4] strains carrying the E6 genotype between 2009 and 2016. Since 2017, the E6 genotype of the NSP4 gene has not been found in any studies. Although only four genes (VP7, VP4, VP6 and NSP4) of the endemic G9P[4] strains in Latin American countries during 2009–2010 were characterized, they showed the same genotypes as those of the Ghanaian and Indian (Kolkata) G9P[4] strains detected during 2011–2016 (G9P[4]-I2-E6). However, the endemic G9P[4] strains in Iran during 2021–2022 showed a different combination of these genes (G9P[4]-I2-E2) [53]. Along with the genome constellation of two recent G9P[4] strains detected in the Czech Republic in 2018 (G9P[4]-I2-E2) [26], it appears that the E6-NSP4 genotype has been replaced by the E2-NSP4 genotype among the endemic G9P[4] strains in several countries. 

A recent study has indicated that after a period of circulation of reassortant RVA strains carrying the DS-1-like backbone, there is a tendency for the reemergence of typical RVA strains (G1/G3-P[8] Wa-like) as the predominant strains [54]. The reappearance and circulation of the G1P[8] strain in fully vaccinated children, who subsequently experience clinically severe diarrhea several years after vaccine introduction, raise concerns about the long-term efficacy of the vaccine. These findings underscore the importance of conducting further monitoring to assess and address any potential impact on vaccine effectiveness.

## 5. Conclusions

The RVA vaccine has proven effective in preventing severe RVA gastroenteritis in children, particularly during the critical first two years of life when the disease is most severe. The widespread implementation of this vaccine has significantly reduced hospital admissions and deaths caused by RVA infections in Ghana and globally. However, it is important to note that the extensive use of the RVA vaccine may have contributed to the emergence and circulation of RVA strains that differ from the genotypes targeted by the vaccine. Our study, along with previous research, has unveiled distinct changes in the distribution patterns of diverse strains, as evidenced by the emergence of RVA genotype constellations in the post-vaccine era. These changes appear to be unique and extend beyond the scope of natural cyclical fluctuations. It is noteworthy that many of the identified reassortant RVA strains carry the DS-1-like backbone, which corresponds to the complete heterotypic genome background of the widely utilized monovalent RVA vaccine strain. Nevertheless, current RVA vaccines have demonstrated their protective efficacy against strains carrying the DS-1-like genome. Reassortant RVA strains, while potentially unstable and circulating for only a brief period, are likely to be replaced by the more typical G1P[8] RVA strains. This detection of G1 RVA in fully vaccinated children presenting with a severe disease several years after vaccination raises concerns about the long-term effectiveness of the vaccine. Consequently, it is crucial to conduct further monitoring to evaluate the potential impact on vaccine effectiveness and to ensure continuous protection against RVA infections.

## Figures and Tables

**Table 1:** Summary of sample collection and proportions of RVA-positive samples in Navrongo, Greater Accra and the Western region (2015–2016).

Age Distribution (Months)	Navrongo		Greater Accra		Western Region		Total	
	Number of AGE Samples	Number of RVA Samples (%)	Number of AGE Samples	Number of RVA Samples (%)	Number of AGE Samples	Number of RVA Samples (%)	Number of AGE Samples	Number of RVA Samples (%)
<6	3	0 (0)	129	26 (20.2)	10	3 (30.0)	**142**	**29 (17.1)**
6–12	30	10 (33.3)	162	36 (22.2)	48	22 (45.8)	**240**	**68 (23.1)**
13–24	25	14 (56.0)	199	43 (21.6)	60	37 (61.7)	**284**	**94 (25.2)**
>24	17	3 (17.6)	93	21 (22.6)	8	2 (25.0)	**118**	**26 (21.2)**
**Total**	**75**	**27 (36.0)**	**583**	**126 (21.6)**	**126**	**64 (50.8)**	**784**	**217 (27.7)**

AGE: acute gastroenteritis. %: The percentage of samples that tested positive for RVA among the collected samples.

## Data Availability

Inquiries regarding data sharing should be directed to the authors.

## Supplementary Materials

The following supporting information can be downloaded at: https://www.mdpi.com/article/10.3390/v15122453/s1, Table S1: The genotype constellations of Ghanaian G9P[4] and G2P[4] strains, as well as those of additional G9P[4] strains for which all 11 genome segments are available in the DNA database; Figure S1: Simplified phylogenetic trees were constructed for 7 genome segments: VP1 (a), VP3 (b), NSP1 (c), NSP2 (d), NSP3 (e), and NSP5 (f). Figure S2: Heat map depicting nucleotide similarity for NSP4 sequences among Ghanaian G9P[4] and others G9P[4] strains. Figure S3: Simplified phylogenetic trees were constructed for 10 genome segments: VP7 (a), VP4 (b), VP6 (c), VP1 (d), VP2 (e), VP3 (f), NSP1 (g), NSP2 (h), NSP3 (i), and NSP5 (k), each with lineage designations for their respective genotypes. Table S2: Distribution of human RVA genotype constellations detected from 1976 to 2006 across 27 countries worldwide.
