# Supplementary material for: Emergence of Intergenogroup Reassortant G9P[4] Strains Following Rotavirus Vaccine Introduction in Ghana
Source: Viruses. 2023 Dec 18;15(12):2453. doi: 10.3390/v15122453 (PMC10747750; doi:10.3390/v15122453)
Supplement: Supplementary file 1 [file viruses-15-02453-s001.zip › 01_Table S1_231218.pdf]

Table S1.The genotype constellations of Ghanaian G9P[4] and G2P[4] strains, as well as those of additional G9P[4] strains for which all 11 genome segments are available in the DNA database.

Major genogroups	VP7	VP4	VP6	VP1	VP2	VP3	NSP1	NSP2	NSP3	NSP4	NSP5
Wa	G1/G3/G4/G9	P[8]	I1	R1	C1	M1	A1	N1	T1	E1	H1
DS-1	G2/G8	P[4]	I2	R2	C2	M2	A2	N2	T2	E2	H2
AU-1	G3	P[9]	I3	R3	C3	M3	A3	N3	T3	E3	H3

	Name of strain	Country of isolation	Age in months	SEX	History of rotavirus vaccine	Year of isolation	The number (%) of segments exhibits similar genotypes to Ghanaian G9P[4] strains	Frequency of appearance	VP7	VP4	VP6	VP1	VP2	VP3	NSP1	NSP2	NSP3	NSP4	NSP5
1	WMH-1444	Ghana	13	F	Yes (Rotarix-2doses)	2016	9 (81%)	Predominant	G2	P[4]	I2	R2	C2	M2	A2	N2	T2	E2	H2
2	WMH-1447	Ghana	20	M	Yes (Rotarix-2doses)	2016	9 (81%)	Predominant	G2	P[4]	I2	R2	C2	M2	A2	N2	T2	E2	H2
3	039M	Ghana	10	F	Yes (Rotarix-2doses)	2015	11 (100%)	Predominant	G9	P[4]	I2	R2	C2	M2	A2	N2	T2	E6	H2
4	082M	Ghana	14	M	Yes (Rotarix-2doses)	2015	11 (100%)	Predominant	G9	P[4]	I2	R2	C2	M2	A2	N2	T2	E6	H2
5	103M	Ghana	17	F	Yes (Rotarix-2doses)	2015	11 (100%)	Predominant	G9	P[4]	I2	R2	C2	M2	A2	N2	T2	E6	H2
6	110M	Ghana	15	M	Yes (Rotarix-2doses)	2015	11 (100%)	Predominant	G9	P[4]	I2	R2	C2	M2	A2	N2	T2	E6	H2
7	119M	Ghana	12	M	Yes (Rotarix-2doses)	2016	11 (100%)	Predominant	G9	P[4]	I2	R2	C2	M2	A2	N2	T2	E6	H2
8	132M	Ghana	22	F	No	2016	11 (100%)	Predominant	G9	P[4]	I2	R2	C2	M2	A2	N2	T2	E6	H2
9	135M	Ghana	20	F	Yes (Rotarix-2doses)	2016	11 (100%)	Predominant	G9	P[4]	I2	R2	C2	M2	A2	N2	T2	E6	H2
10	WMH-1439	Ghana	30	unknow	Yes (Rotarix-2doses)	2015	11 (100%)	Predominant	G9	P[4]	I2	R2	C2	M2	A2	N2	T2	E6	H2
11	WMH-1441	Ghana	25	M	Yes (Rotarix-2doses)	2015	11 (100%)	Predominant	G9	P[4]	I2	R2	C2	M2	A2	N2	T2	E6	H2
12	WMH-1445	Ghana	12	M	Yes (Rotarix-2doses)	2016	11 (100%)	Predominant	G9	P[4]	I2	R2	C2	M2	A2	N2	T2	E6	H2
13	WMH-1446	Ghana	30	M	Yes (Rotarix-2doses)	2016	11 (100%)	Predominant	G9	P[4]	I2	R2	C2	M2	A2	N2	T2	E6	H2
14	WMH-1451	Ghana	20	F	Yes (Rotarix-2doses)	2016	11 (100%)	Predominant	G9	P[4]	I2	R2	C2	M2	A2	N2	T2	E6	H2
15	WMH-1452	Ghana	23	F	Yes (Rotarix-2doses)	2016	11 (100%)	Predominant	G9	P[4]	I2	R2	C2	M2	A2	N2	T2	E6	H2
16	WMH-1453	Ghana	7	F	Yes (Rotarix-2doses)	2016	11 (100%)	Predominant	G9	P[4]	I2	R2	C2	M2	A2	N2	T2	E6	H2
17	EUHC-002	Ghana	16	M	unknown	2016	11 (100%)	Predominant	G9	P[4]	I2	R2	C2	M2	A2	N2	T2	E6	H2
19	1157A	Paraguay				2007	10 (90%)	Sporadic	G9	P[4]	I2	R2	C2	M2	A2	N2	T2	E3	H2
20	Bang-068	Bangladesh				2008	10 (90%)	Sporadic	G9	P[4]	I2	R2	C2	M2	A2	N2	T2	E2	H2

21	Seoul1622	South Korea	2011	4 (36 %)	Sporadic	G9	P[4]	I2	R2	C1	M1	A1	N1	T1	E1	H1
22	RV09	Pune, India	2009	9 (81%)	Predominant	G9	P[4]	I2	R2	C2	M2	A2	N2	T1	E2	H2
18	LB1562	USA	2010	11 (100%)	Sporadic	G9	P[4]	I2	R2	C2	M2	A2	N2	T2	E6	H2
23	RV10	Pune, India	2010	11 (100%)	Predominant	G9	P[4]	I2	R2	C2	M2	A2	N2	T2	E6	H2
24	RV11	Pune, India	2011	11 (100%)	Predominant	G9	P[4]	I2	R2	C2	M2	A2	N2	T2	E6	H2
25	Kol-006	Kolkata, India	2011	10 (90%)	Predominant	G9	P[4]	I2	R2	C2	M2	A2	N1	T2	E6	H2
26	Kol-018	Kolkata, India	2011	10 (90%)	Predominant	G9	P[4]	I2	R2	C2	M2	A2	N1	T2	E6	H2
27	Kol-028	Kolkata, India	2012	11 (100%)	Predominant	G9	P[4]	I2	R2	C2	M2	A2	N2	T2	E6	H2
28	Kol-040	Kolkata, India	2012	11 (100%)	Predominant	G9	P[4]	I2	R2	C2	M2	A2	N2	T2	E6	H2
29	Kol-041	Kolkata, India	2012	11 (100%)	Predominant	G9	P[4]	I2	R2	C2	M2	A2	N2	T2	E6	H2
30	Kol-063	Kolkata, India	2012	11 (100%)	Predominant	G9	P[4]	I2	R2	C2	M2	A2	N2	T2	E6	H2
31	S120088	Japan	2012	10 (90%)	Sporadic	G9	P[4]	I2	R2	C2	M2	A2	N2	T1	E2	H2
32	11584	Japan	2013	10 (90%)	Sporadic	G9	P[4]	I2	R2	C2	M2	A2	N2	T2	E2	H2
33	MRC-DPRU566	Rwanda	2013	7 (63%)	Sporadic	G9	P[4]	I2	R2	C2	M2	A1	N1	T1	E1	H1
34	Kol-047	Kolkata, India	2013	10 (90%)	Predominant	G9	P[4]	I2	R2	C2	M2	A2	N1	T2	E6	H2
35	Kol-051	Kolkata, India	2013	10 (90%)	Predominant	G9	P[4]	I2	R2	C2	M2	A2	N1	T2	E6	H2
36	Kol-064	Kolkata, India	2013	10 (90%)	Predominant	G9	P[4]	I2	R2	C2	M2	A2	N1	T2	E6	H2
37	Kol-065	Kolkata, India	2013	10 (90%)	Predominant	G9	P[4]	I2	R2	C2	M2	A2	N1	T2	E6	H2
38	Kol-066	Kolkata, India	2013	10 (90%)	Predominant	G9	P[4]	I2	R2	C2	M2	A2	N1	T2	E6	H2
39	AN18	Italy	2016	10 (90%)	Sporadic	G9	P[4]	I2	R2	C2	M2	A2	N2	T2	E2	H2
40	AN19	Italy	2016	10 (90%)	Sporadic	G9	P[4]	I2	R2	C2	M2	A2	N2	T2	E2	H2
41	AN22	Italy	2016	10 (90%)	Sporadic	G9	P[4]	I2	R2	C2	M2	A2	N2	T2	E2	H2
42	H186	Czech Republic	2018	9 (81%)	Sporadic	G9	P[4]	I2	R2	C2	M2	A2	N1	T2	E2	H2
43	H187	Czech Republic	2018	9 (81%)	Sporadic	G9	P[4]	I2	R2	C2	M2	A2	N1	T2	E2	H2

The strains identified in this study are highlighted in red
