# Supplementary material for: Emergence of Intergenogroup Reassortant G9P[4] Strains Following Rotavirus Vaccine Introduction in Ghana
Source: Viruses. 2023 Dec 18;15(12):2453. doi: 10.3390/v15122453 (PMC10747750; doi:10.3390/v15122453)

Figure S1: Simplified phylogenetic trees were constructed for 7 genome segments: VP1 (a), VP3 (b), NSP1 (c), NSP2 (d), NSP3 (e), and NSP5 (f). The tree includes the 15 Ghanaian G9P[4] strains from this study (indicated by red dots), as well as additional G9P[4] strains (highlighted by red branch trees). Additionally, two Ghanaian G2P[4] strains from this study, along with Ghanaian strains carrying genotype 2 for these 7 genome segments, were incorporated into the analysis (indicated by blue dots). Global reference RVAs carrying genotype 2 for these genome segments were also integrated into the trees. The trees were generated using the maximum likelihood method within the MEGA software package (version 6), with bootstrap values determined from 1,000 replicate trials. Genetic distances are indicated at the bottom, and percent bootstrap support is displayed at each node when it reaches 70% or higher.

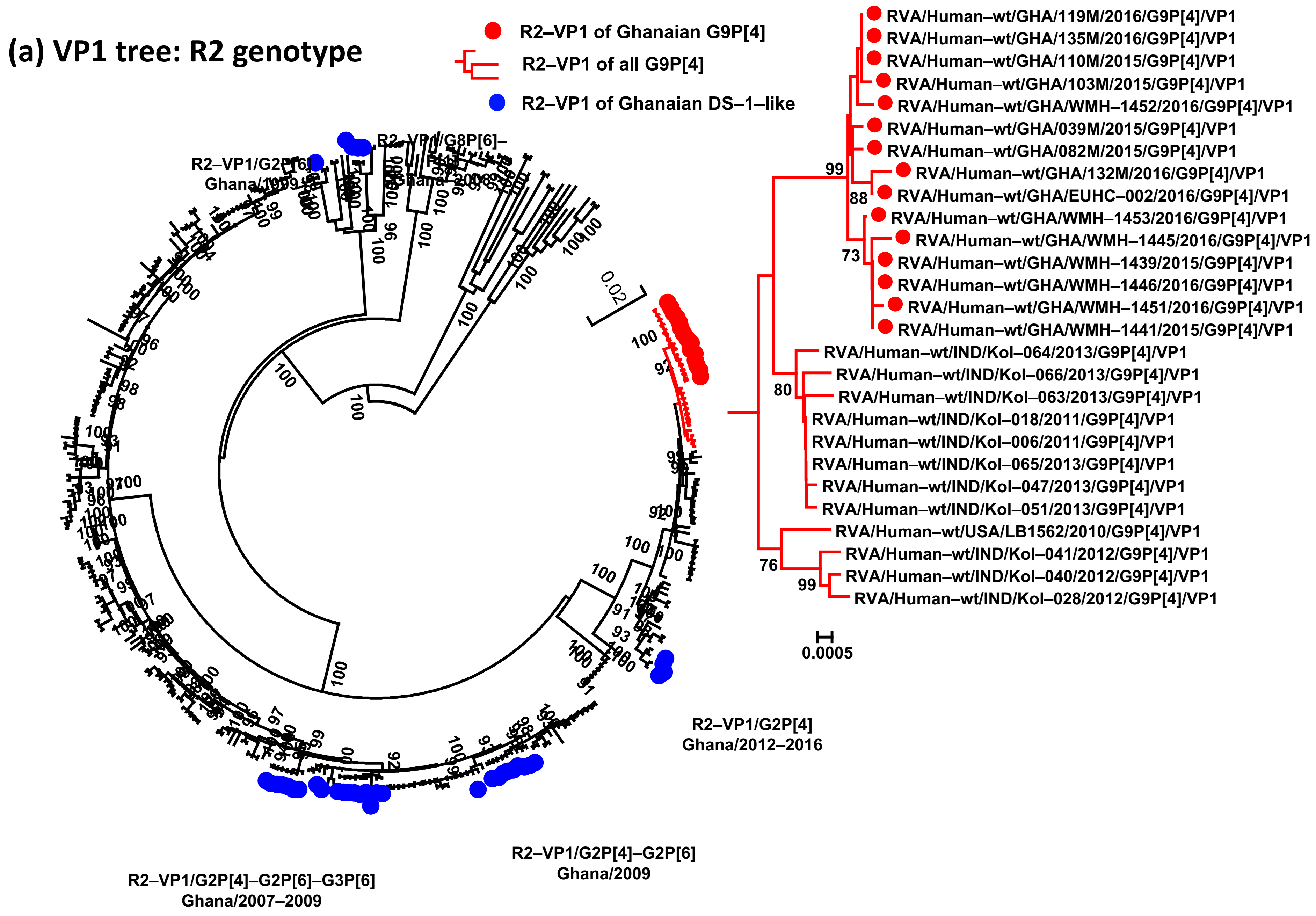

(a) VP1 tree: R2 genotype
R2–VP1 of Ghanaian G9P[4]
R2–VP1 of all G9P[4]
R2–VP1 of Ghanaian DS–1–like
R2–VP1/G2P[4]
Ghana/2012–2016
R2–VP1/G2P[4]–G2P[6]
Ghana/2009
R2–VP1/G2P[4]–G2P[6]–G3P[6]
Ghana/2007–2009
0.02
RVA/Human–wt/GHA/119M/2016/G9P[4]/VP1
RVA/Human–wt/GHA/135M/2016/G9P[4]/VP1
RVA/Human–wt/GHA/110M/2015/G9P[4]/VP1
RVA/Human–wt/GHA/103M/2015/G9P[4]/VP1
RVA/Human–wt/GHA/WMH–1452/2016/G9P[4]/VP1
RVA/Human–wt/GHA/039M/2015/G9P[4]/VP1
RVA/Human–wt/GHA/082M/2015/G9P[4]/VP1
RVA/Human–wt/GHA/132M/2016/G9P[4]/VP1
RVA/Human–wt/GHA/EUHC–002/2016/G9P[4]/VP1
RVA/Human–wt/GHA/WMH–1453/2016/G9P[4]/VP1
RVA/Human–wt/GHA/WMH–1445/2016/G9P[4]/VP1
RVA/Human–wt/GHA/WMH–1439/2015/G9P[4]/VP1
RVA/Human–wt/GHA/WMH–1446/2016/G9P[4]/VP1
RVA/Human–wt/GHA/WMH–1451/2016/G9P[4]/VP1
RVA/Human–wt/GHA/WMH–1441/2015/G9P[4]/VP1
RVA/Human–wt/IND/Kol–064/2013/G9P[4]/VP1
RVA/Human–wt/IND/Kol–066/2013/G9P[4]/VP1
RVA/Human–wt/IND/Kol–063/2013/G9P[4]/VP1
RVA/Human–wt/IND/Kol–018/2011/G9P[4]/VP1
RVA/Human–wt/IND/Kol–006/2011/G9P[4]/VP1
RVA/Human–wt/IND/Kol–065/2013/G9P[4]/VP1
RVA/Human–wt/IND/Kol–047/2013/G9P[4]/VP1
RVA/Human–wt/IND/Kol–051/2013/G9P[4]/VP1
RVA/Human–wt/USA/LB1562/2010/G9P[4]/VP1
RVA/Human–wt/IND/Kol–041/2012/G9P[4]/VP1
RVA/Human–wt/IND/Kol–040/2012/G9P[4]/VP1
RVA/Human–wt/IND/Kol–028/2012/G9P[4]/VP1
99
88
73
80
76
99
0.0005

(b) VP3 tree: M2 genotype

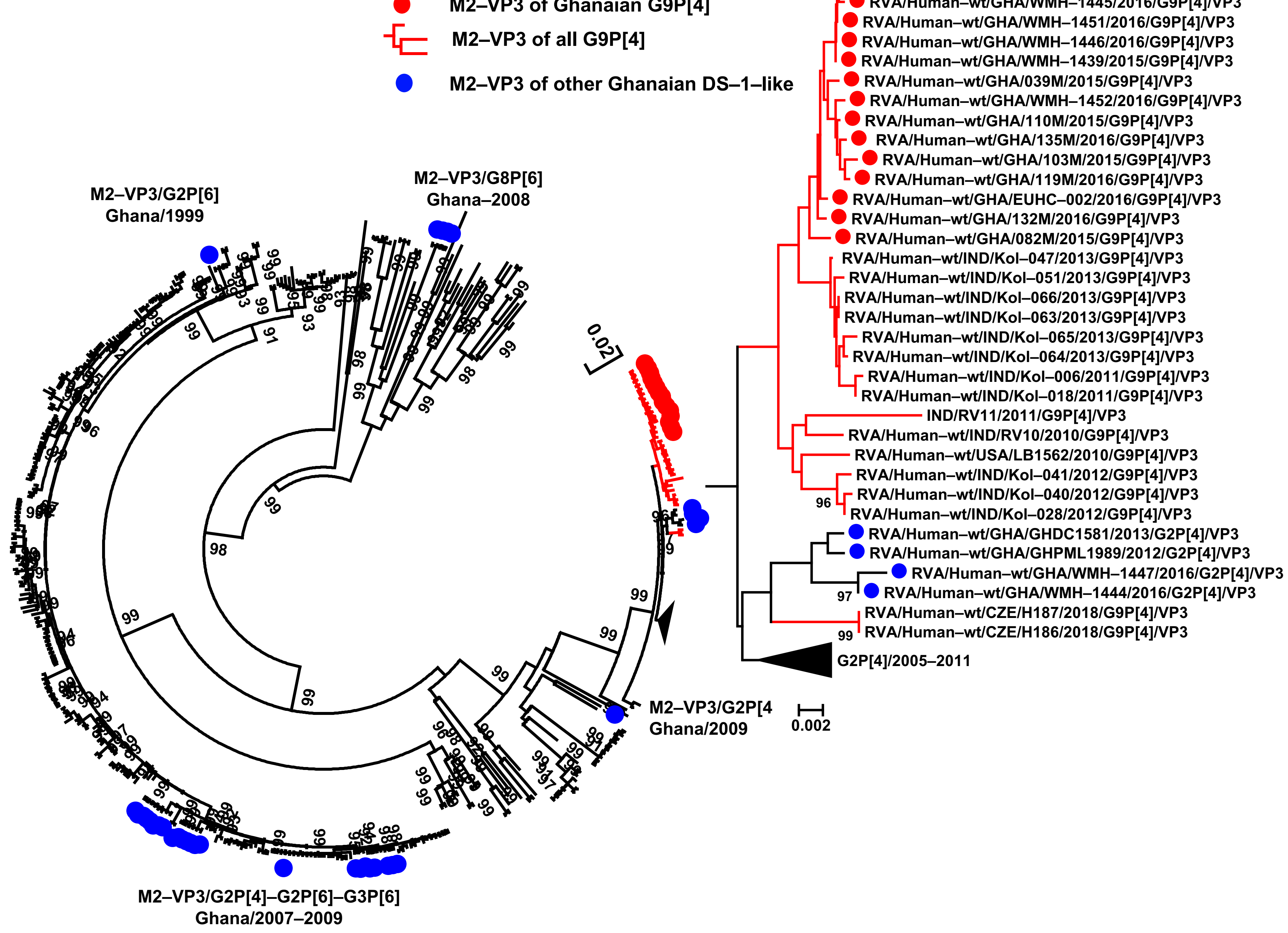

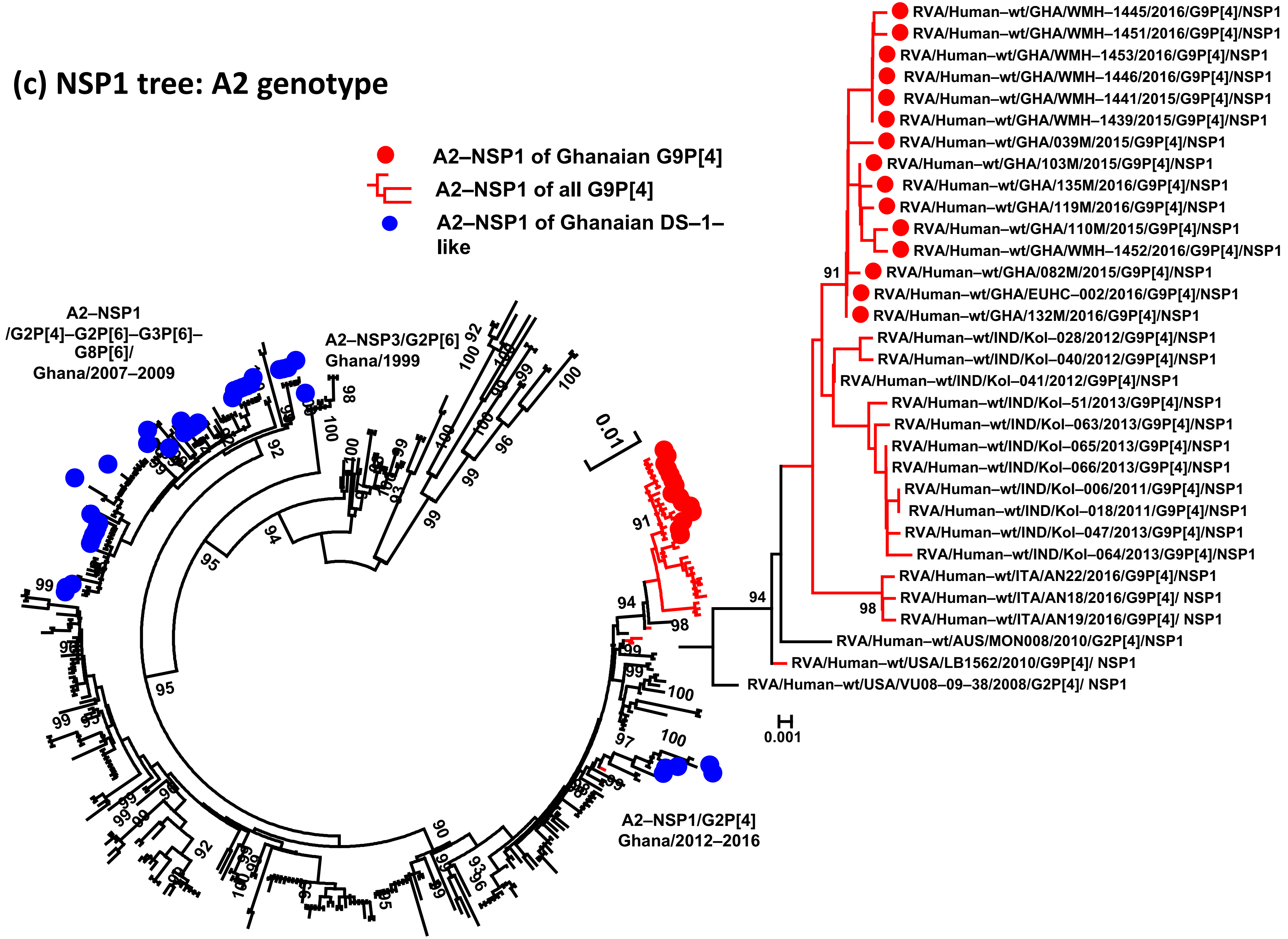
(c) NSP1 tree: A2 genotype
A2–NSP1 of Ghanaian G9P[4]
A2–NSP1 of all G9P[4]
A2–NSP1 of Ghanaian DS–1–like
A2–NSP1 /G2P[4]–G2P[6]–G3P[6]–G8P[6]/ Ghana/2007–2009
A2–NSP3/G2P[6] Ghana/1999
A2–NSP1/G2P[4] Ghana/2012–2016
0.01
0.001
RVA/Human–wt/GHA/WMH–1445/2016/G9P[4]/NSP1
RVA/Human–wt/GHA/WMH–1451/2016/G9P[4]/NSP1
RVA/Human–wt/GHA/WMH–1453/2016/G9P[4]/NSP1
RVA/Human–wt/GHA/WMH–1446/2016/G9P[4]/NSP1
RVA/Human–wt/GHA/WMH–1441/2015/G9P[4]/NSP1
RVA/Human–wt/GHA/WMH–1439/2015/G9P[4]/NSP1
RVA/Human–wt/GHA/039M/2015/G9P[4]/NSP1
RVA/Human–wt/GHA/103M/2015/G9P[4]/NSP1
RVA/Human–wt/GHA/135M/2016/G9P[4]/NSP1
RVA/Human–wt/GHA/119M/2016/G9P[4]/NSP1
RVA/Human–wt/GHA/110M/2015/G9P[4]/NSP1
RVA/Human–wt/GHA/WMH–1452/2016/G9P[4]/NSP1
RVA/Human–wt/GHA/082M/2015/G9P[4]/NSP1
RVA/Human–wt/GHA/EUHC–002/2016/G9P[4]/NSP1
RVA/Human–wt/GHA/132M/2016/G9P[4]/NSP1
RVA/Human–wt/IND/Kol–028/2012/G9P[4]/NSP1
RVA/Human–wt/IND/Kol–040/2012/G9P[4]/NSP1
RVA/Human–wt/IND/Kol–041/2012/G9P[4]/NSP1
RVA/Human–wt/IND/Kol–51/2013/G9P[4]/NSP1
RVA/Human–wt/IND/Kol–063/2013/G9P[4]/NSP1
RVA/Human–wt/IND/Kol–065/2013/G9P[4]/NSP1
RVA/Human–wt/IND/Kol–066/2013/G9P[4]/NSP1
RVA/Human–wt/IND/Kol–006/2011/G9P[4]/NSP1
RVA/Human–wt/IND/Kol–018/2011/G9P[4]/NSP1
RVA/Human–wt/IND/Kol–047/2013/G9P[4]/NSP1
RVA/Human–wt/IND/Kol–064/2013/G9P[4]/NSP1
RVA/Human–wt/ITA/AN22/2016/G9P[4]/NSP1
RVA/Human–wt/ITA/AN18/2016/G9P[4]/ NSP1
RVA/Human–wt/ITA/AN19/2016/G9P[4]/ NSP1
RVA/Human–wt/AUS/MON008/2010/G2P[4]/NSP1
RVA/Human–wt/USA/LB1562/2010/G9P[4]/ NSP1
RVA/Human–wt/USA/VU08–09–38/2008/G2P[4]/ NSP1
91
94
98

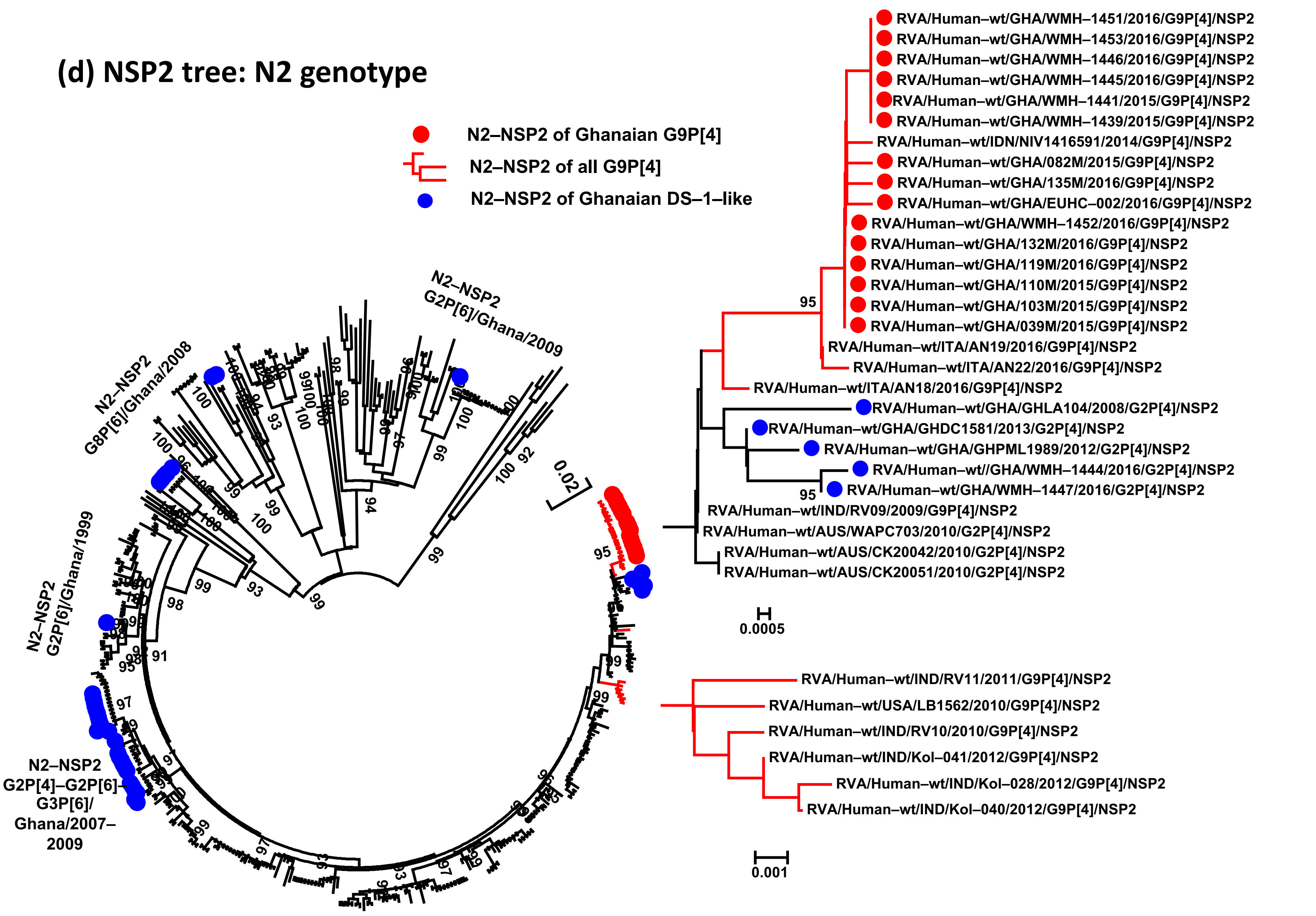
(d) NSP2 tree: N2 genotype
N2–NSP2 of Ghanaian G9P[4]
N2–NSP2 of all G9P[4]
N2–NSP2 of Ghanaian DS–1–like
N2–NSP2 G2P[6]/Ghana/2009
N2–NSP2 G8P[6]/Ghana/2008
N2–NSP2 G2P[6]/Ghana/1999
N2–NSP2 G2P[4]–G2P[6]–G3P[6]/ Ghana/2007–2009
0.02
RVA/Human–wt/GHA/WMH–1451/2016/G9P[4]/NSP2
RVA/Human–wt/GHA/WMH–1453/2016/G9P[4]/NSP2
RVA/Human–wt/GHA/WMH–1446/2016/G9P[4]/NSP2
RVA/Human–wt/GHA/WMH–1445/2016/G9P[4]/NSP2
RVA/Human–wt/GHA/WMH–1441/2015/G9P[4]/NSP2
RVA/Human–wt/GHA/WMH–1439/2015/G9P[4]/NSP2
RVA/Human–wt/IDN/NIV1416591/2014/G9P[4]/NSP2
RVA/Human–wt/GHA/082M/2015/G9P[4]/NSP2
RVA/Human–wt/GHA/135M/2016/G9P[4]/NSP2
RVA/Human–wt/GHA/EUHC–002/2016/G9P[4]/NSP2
RVA/Human–wt/GHA/WMH–1452/2016/G9P[4]/NSP2
RVA/Human–wt/GHA/132M/2016/G9P[4]/NSP2
RVA/Human–wt/GHA/119M/2016/G9P[4]/NSP2
RVA/Human–wt/GHA/110M/2015/G9P[4]/NSP2
RVA/Human–wt/GHA/103M/2015/G9P[4]/NSP2
RVA/Human–wt/GHA/039M/2015/G9P[4]/NSP2
RVA/Human–wt/ITA/AN19/2016/G9P[4]/NSP2
RVA/Human–wt/ITA/AN22/2016/G9P[4]/NSP2
RVA/Human–wt/ITA/AN18/2016/G9P[4]/NSP2
RVA/Human–wt/GHA/GHLA104/2008/G2P[4]/NSP2
RVA/Human–wt/GHA/GHDC1581/2013/G2P[4]/NSP2
RVA/Human–wt/GHA/GHPML1989/2012/G2P[4]/NSP2
RVA/Human–wt//GHA/WMH–1444/2016/G2P[4]/NSP2
RVA/Human–wt/GHA/WMH–1447/2016/G2P[4]/NSP2
RVA/Human–wt/IND/RV09/2009/G9P[4]/NSP2
RVA/Human–wt/AUS/WAPC703/2010/G2P[4]/NSP2
RVA/Human–wt/AUS/CK20042/2010/G2P[4]/NSP2
RVA/Human–wt/AUS/CK20051/2010/G2P[4]/NSP2
0.0005
RVA/Human–wt/IND/RV11/2011/G9P[4]/NSP2
RVA/Human–wt/USA/LB1562/2010/G9P[4]/NSP2
RVA/Human–wt/IND/RV10/2010/G9P[4]/NSP2
RVA/Human–wt/IND/Kol–041/2012/G9P[4]/NSP2
RVA/Human–wt/IND/Kol–028/2012/G9P[4]/NSP2
RVA/Human–wt/IND/Kol–040/2012/G9P[4]/NSP2
0.001

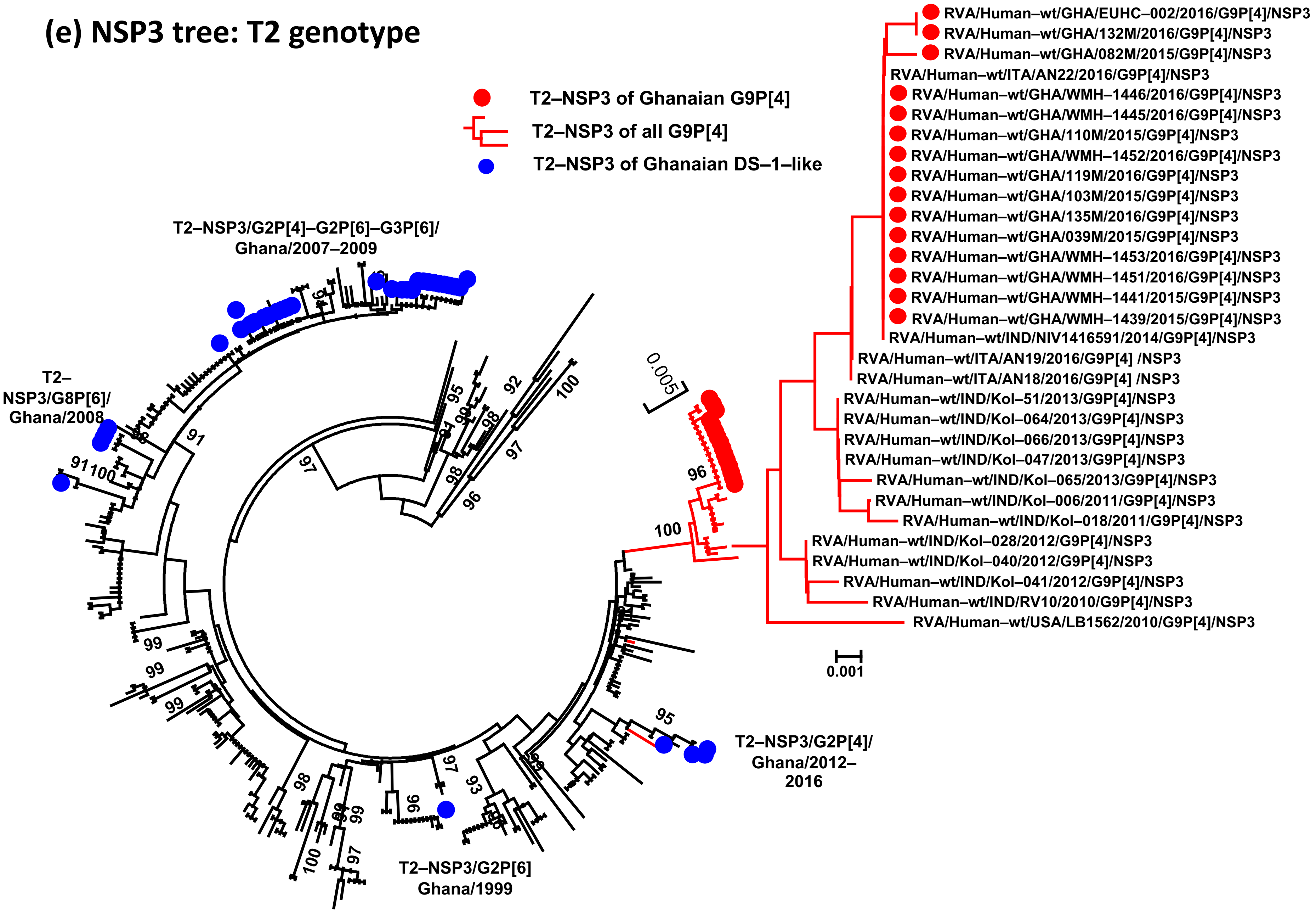

(e) NSP3 tree: T2 genotype
T2–NSP3 of Ghanaian G9P[4]
T2–NSP3 of all G9P[4]
T2–NSP3 of Ghanaian DS–1–like
T2–NSP3/G2P[4]–G2P[6]–G3P[6]/
Ghana/2007–2009
T2–
NSP3/G8P[6]/
Ghana/2008
T2–NSP3/G2P[4]/
Ghana/2012–
2016
T2–NSP3/G2P[6]
Ghana/1999
0.005
RVA/Human–wt/GHA/EUHC–002/2016/G9P[4]/NSP3
RVA/Human–wt/GHA/132M/2016/G9P[4]/NSP3
RVA/Human–wt/GHA/082M/2015/G9P[4]/NSP3
RVA/Human–wt/ITA/AN22/2016/G9P[4]/NSP3
RVA/Human–wt/GHA/WMH–1446/2016/G9P[4]/NSP3
RVA/Human–wt/GHA/WMH–1445/2016/G9P[4]/NSP3
RVA/Human–wt/GHA/110M/2015/G9P[4]/NSP3
RVA/Human–wt/GHA/WMH–1452/2016/G9P[4]/NSP3
RVA/Human–wt/GHA/119M/2016/G9P[4]/NSP3
RVA/Human–wt/GHA/103M/2015/G9P[4]/NSP3
RVA/Human–wt/GHA/135M/2016/G9P[4]/NSP3
RVA/Human–wt/GHA/039M/2015/G9P[4]/NSP3
RVA/Human–wt/GHA/WMH–1453/2016/G9P[4]/NSP3
RVA/Human–wt/GHA/WMH–1451/2016/G9P[4]/NSP3
RVA/Human–wt/GHA/WMH–1441/2015/G9P[4]/NSP3
RVA/Human–wt/GHA/WMH–1439/2015/G9P[4]/NSP3
RVA/Human–wt/IND/NIV1416591/2014/G9P[4]/NSP3
RVA/Human–wt/ITA/AN19/2016/G9P[4] /NSP3
RVA/Human–wt/ITA/AN18/2016/G9P[4] /NSP3
RVA/Human–wt/IND/Kol–51/2013/G9P[4]/NSP3
RVA/Human–wt/IND/Kol–064/2013/G9P[4]/NSP3
RVA/Human–wt/IND/Kol–066/2013/G9P[4]/NSP3
RVA/Human–wt/IND/Kol–047/2013/G9P[4]/NSP3
RVA/Human–wt/IND/Kol–065/2013/G9P[4]/NSP3
RVA/Human–wt/IND/Kol–006/2011/G9P[4]/NSP3
RVA/Human–wt/IND/Kol–018/2011/G9P[4]/NSP3
RVA/Human–wt/IND/Kol–028/2012/G9P[4]/NSP3
RVA/Human–wt/IND/Kol–040/2012/G9P[4]/NSP3
RVA/Human–wt/IND/Kol–041/2012/G9P[4]/NSP3
RVA/Human–wt/IND/RV10/2010/G9P[4]/NSP3
RVA/Human–wt/USA/LB1562/2010/G9P[4]/NSP3
0.001

(f) NSP5 tree: H2 genotype

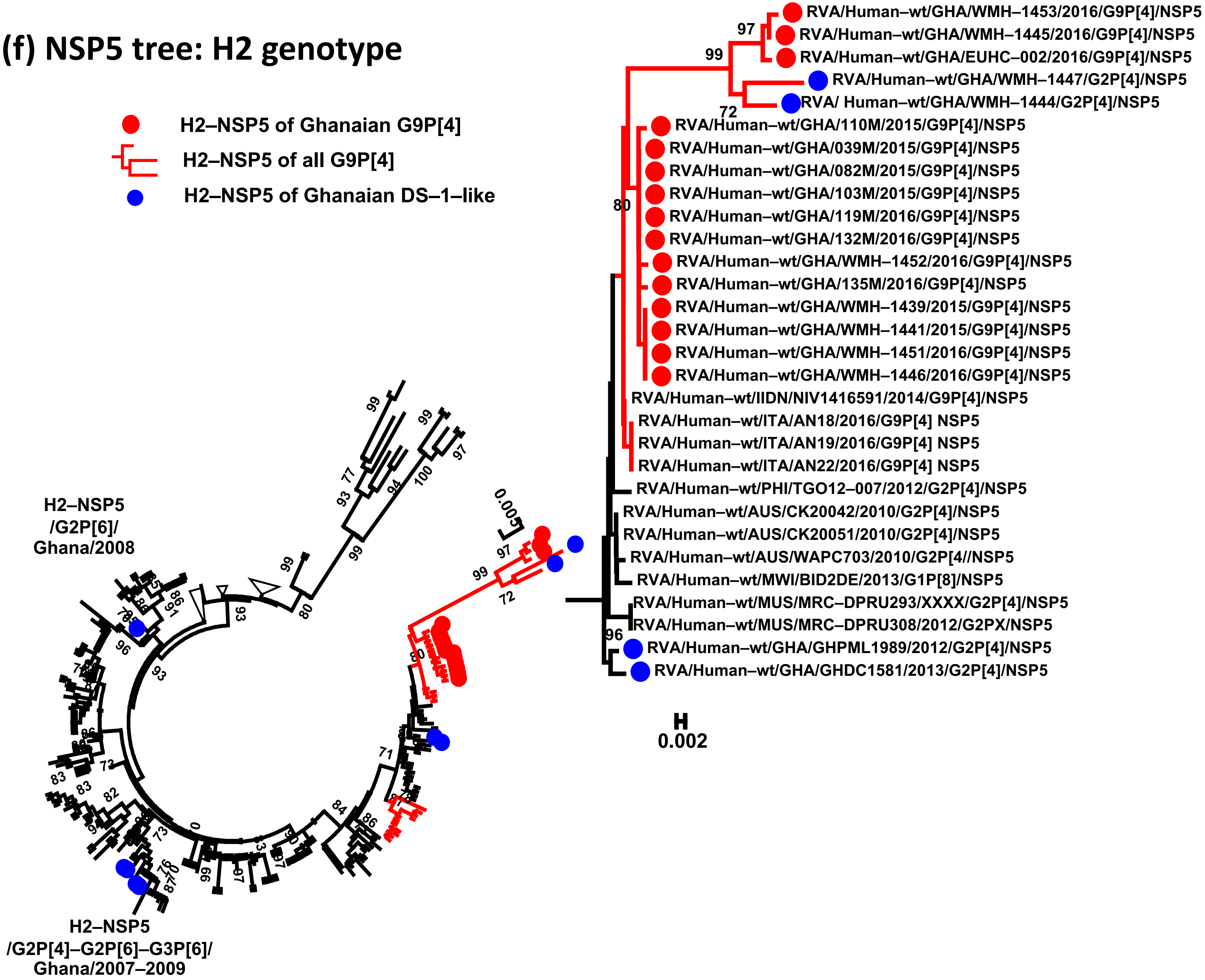

Supplement: Supplementary file 1 [file viruses-15-02453-s001.zip › 02_Figure S1_231218.pdf]
