# Supplementary material for: Emergence of Intergenogroup Reassortant G9P[4] Strains Following Rotavirus Vaccine Introduction in Ghana
Source: Viruses. 2023 Dec 18;15(12):2453. doi: 10.3390/v15122453 (PMC10747750; doi:10.3390/v15122453)

Figure S2: Heat map depicting nucleotide similarity for NSP4 sequences among Ghanaian G9P[4] and others G9P[4] strains.

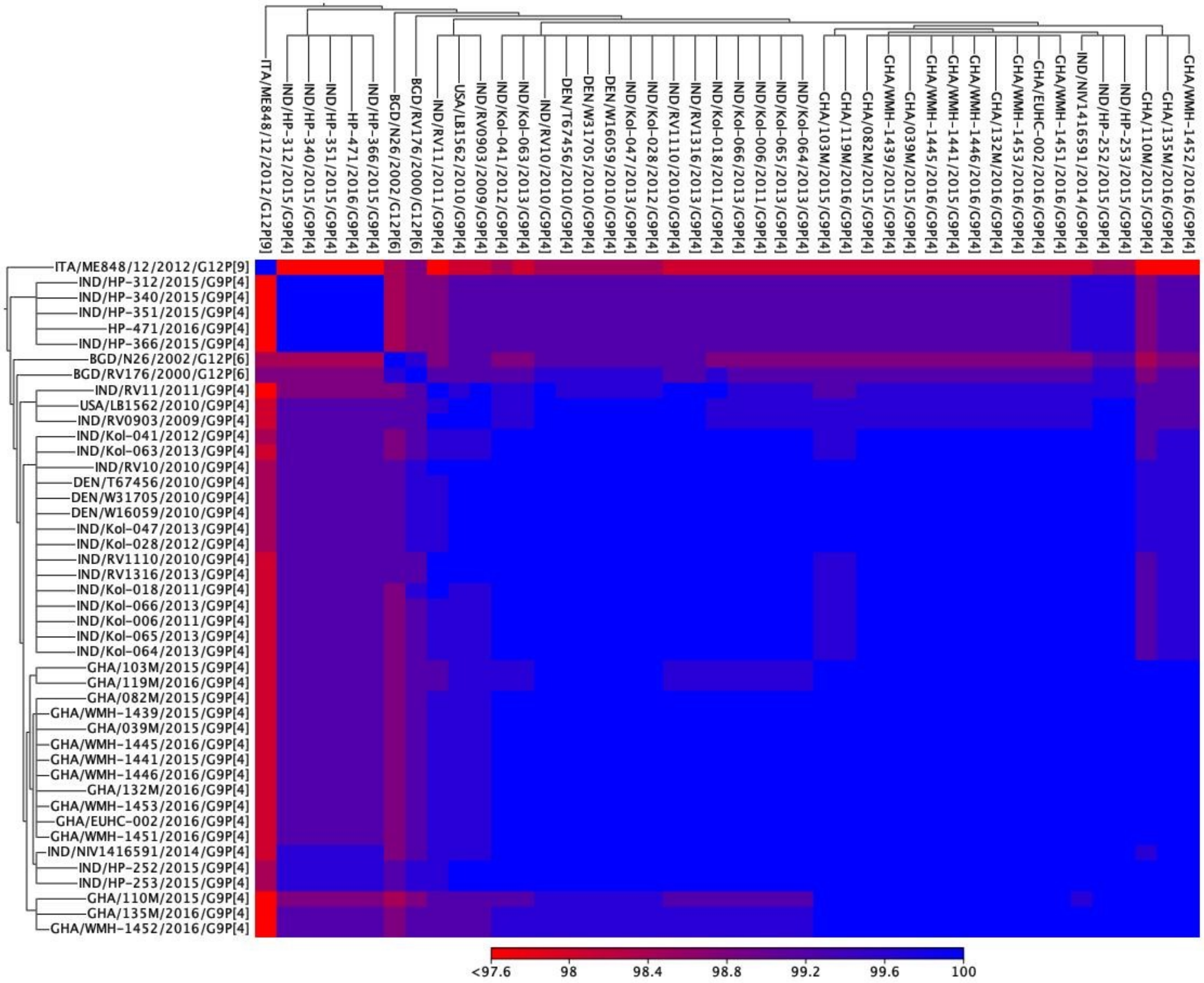

Supplement: Supplementary file 1 [file viruses-15-02453-s001.zip › 03_Figure S2 231218.pdf]
