# Supplementary material for: Emergence of Intergenogroup Reassortant G9P[4] Strains Following Rotavirus Vaccine Introduction in Ghana
Source: Viruses. 2023 Dec 18;15(12):2453. doi: 10.3390/v15122453 (PMC10747750; doi:10.3390/v15122453)

Figure S3. Simplified phylogenetic trees were constructed for 10 genome segments: VP7 (a), VP4 (b), VP6 (c), VP1 (d), VP2 (e), VP3(f), NSP1 (g), NSP2(h), NSP3(i), and NSP5(j), each with lineage designations for their respective genotypes. The tree includes the 15 Ghanaian G9P[4] strains from this study (indicated by red dots), as well as additional G9P[4] strains (indicated by red branch trees). Furthermore, two Ghanaian G2P[4] strains from this study, along with Ghanaian strains carrying G9/VP7, P[4]/VP4 and genotype 2 for other genome segments, were incorporated into the analysis (indicated by blue dots). Global reference RVAs carrying G9/VP7, P[4]/VP4 and genotype 2 for other genome segments were also integrated into the trees. Lineage designations are based on the recently proposed classification for genotype 2 of globally circulating DS-1-like strains by Agbemabiese et al., 2019 [30], and for genotypes 2 and 9 for VP7 genes by Doan et al., 2015 and 2017 [20, 33]. The trees are generated using the maximum likelihood method within the MEGA software package (version 6), with bootstrap values determined from 1,000 replicate trials. Genetic distances are indicated at the bottom, and percent bootstrap support is displayed at each node when it reaches 70% or higher. The tree topologies are presented in a rectangular style.

(a) VP7 tree (Rectangular style): G9 genotype

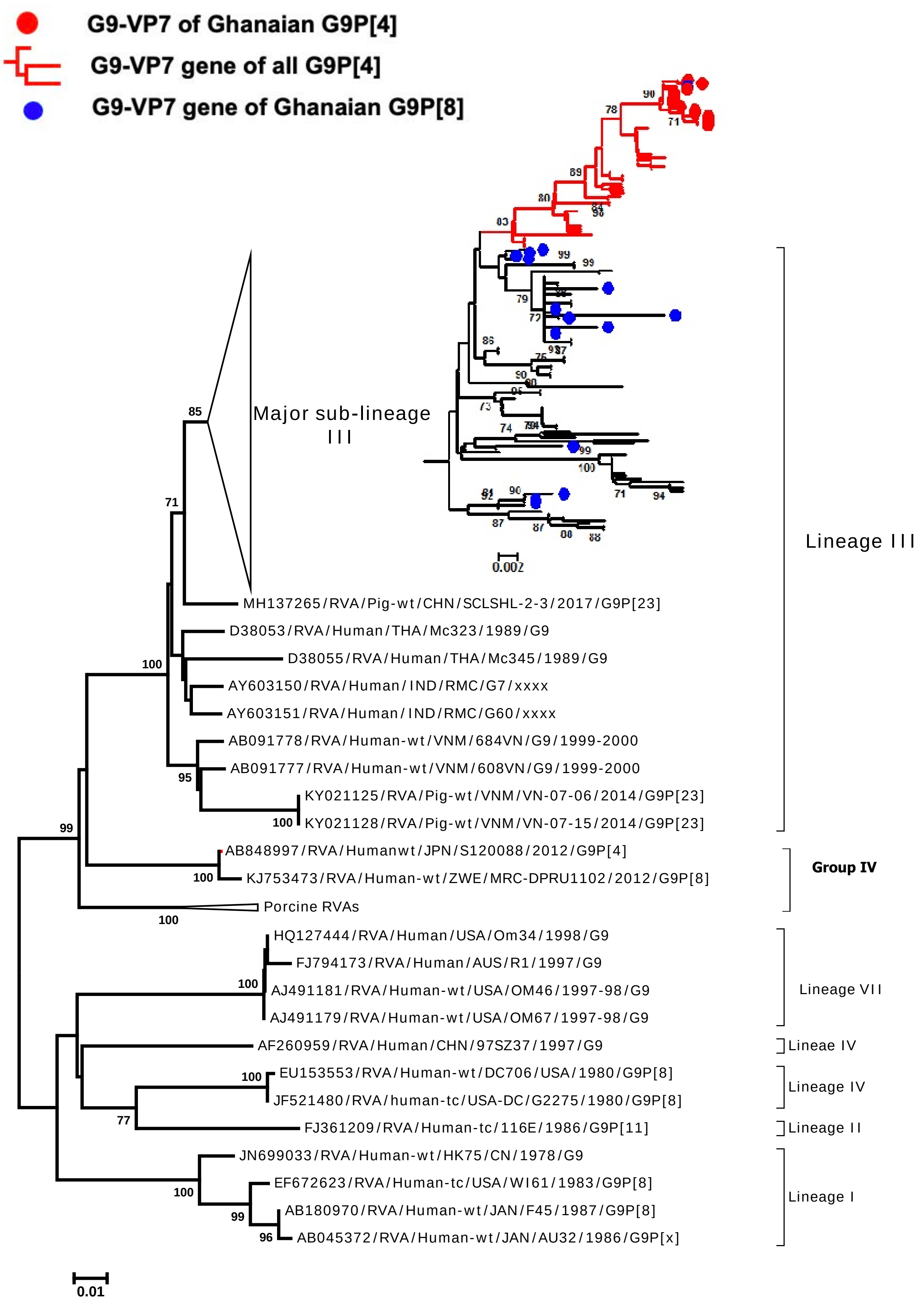

(b) VP4 tree (Rectangular style): P[4] genotype

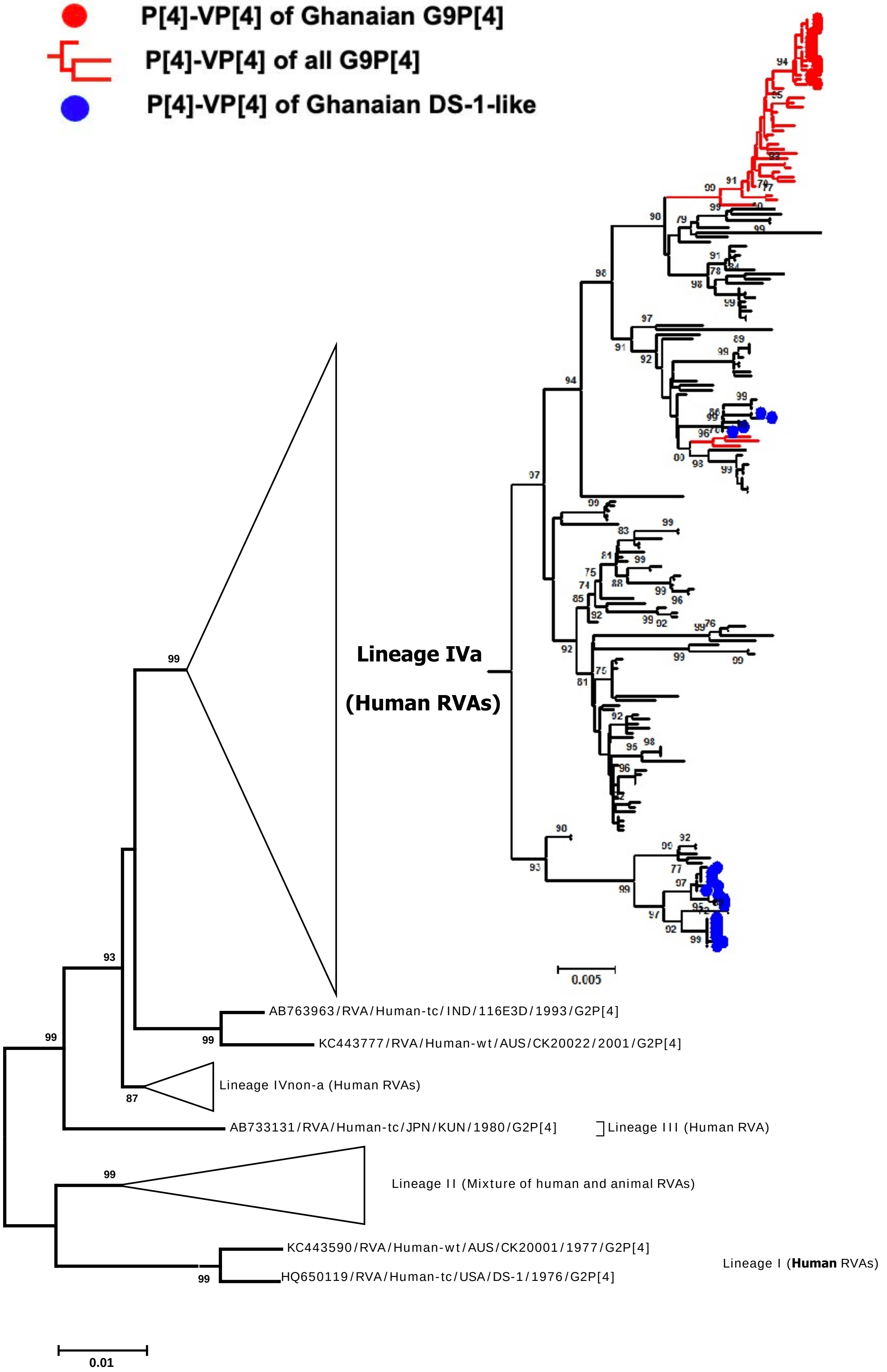

(c) VP6 tree (Rectangular style): I2 genotype

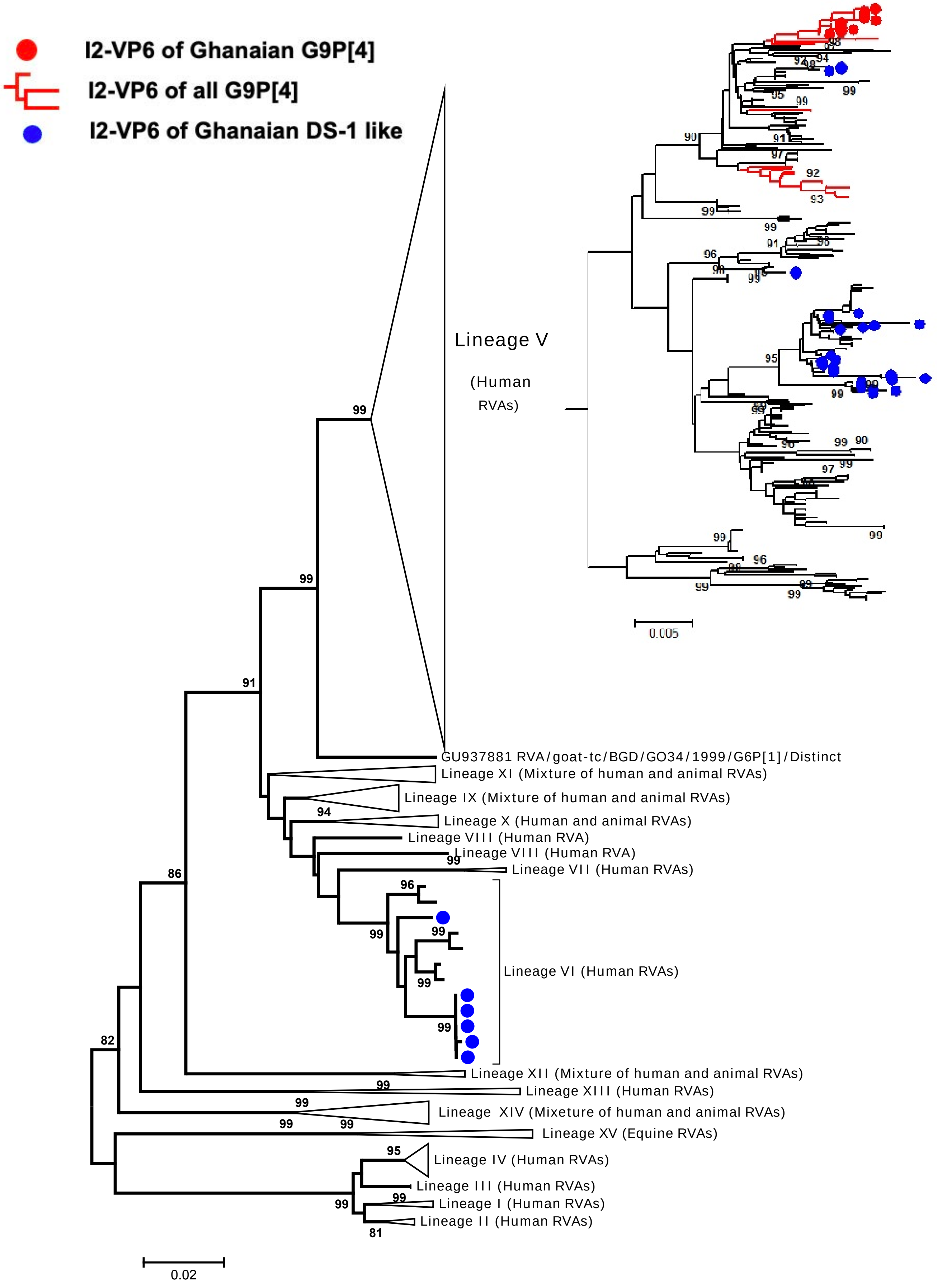

(d) VP1 tree (Rectangular style): R2 genotype

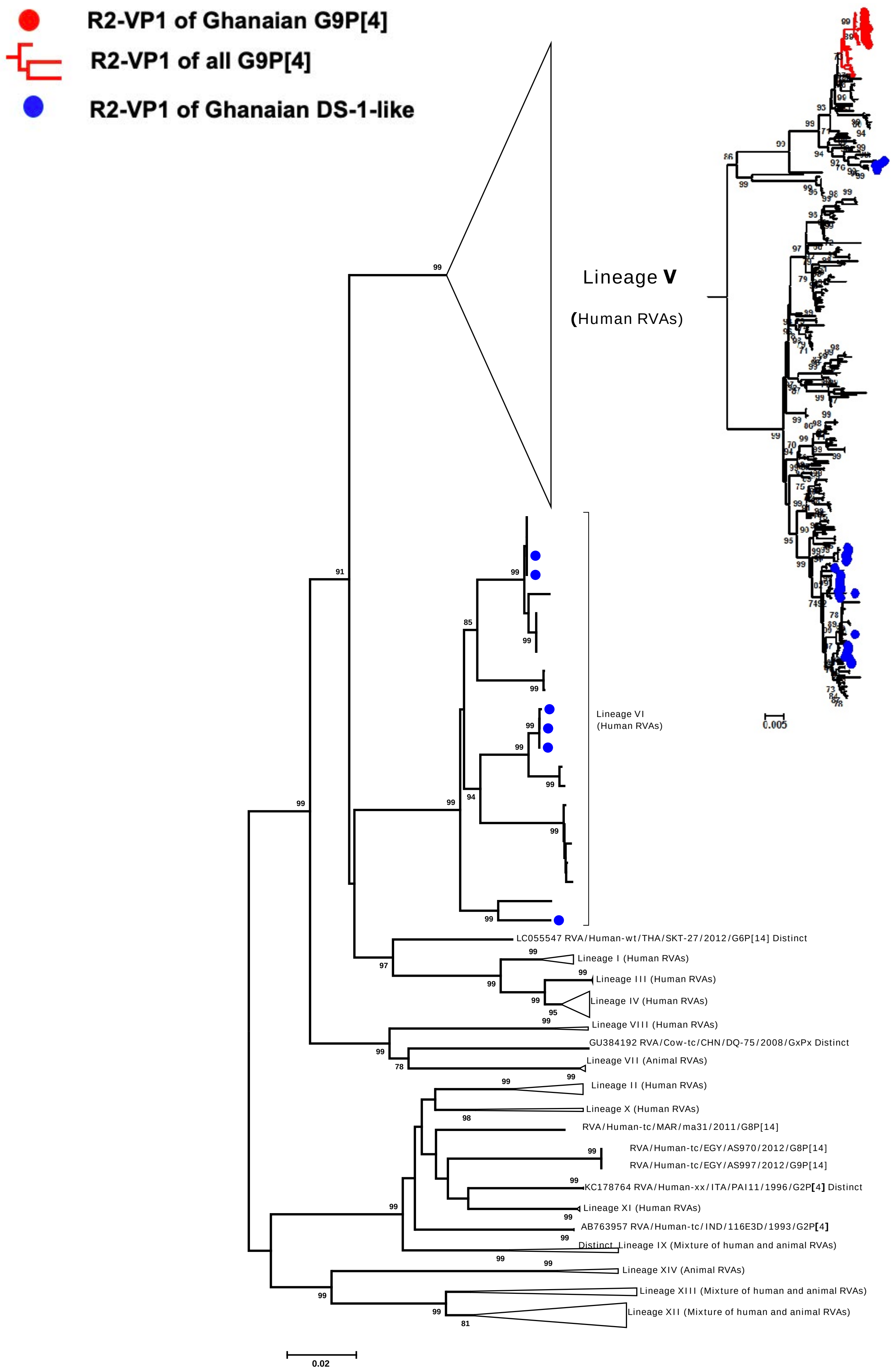

(e) VP2 tree (Rectangular style): C2 genotype

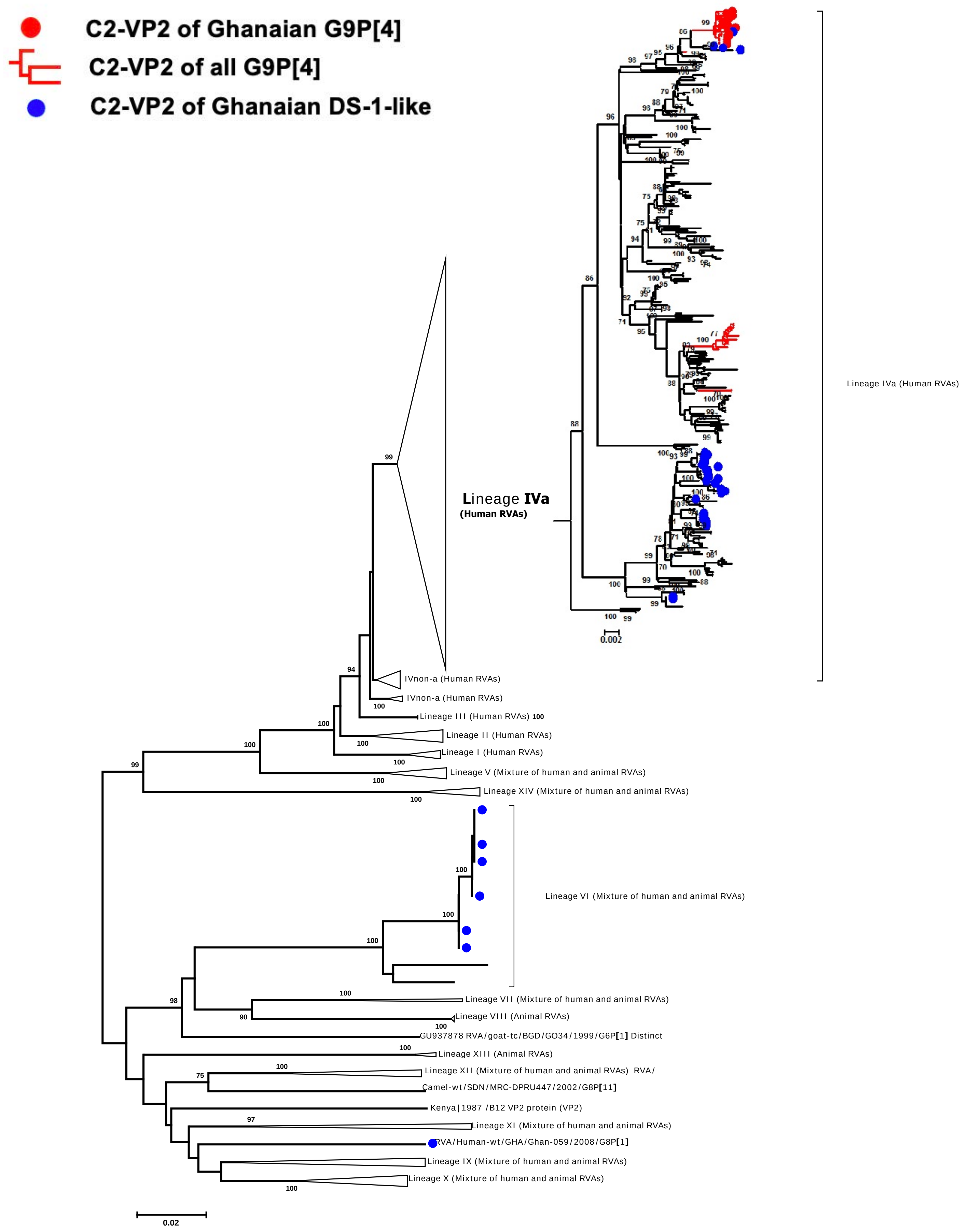

(f)VP3 tree (Rectangular style): M2 genotype

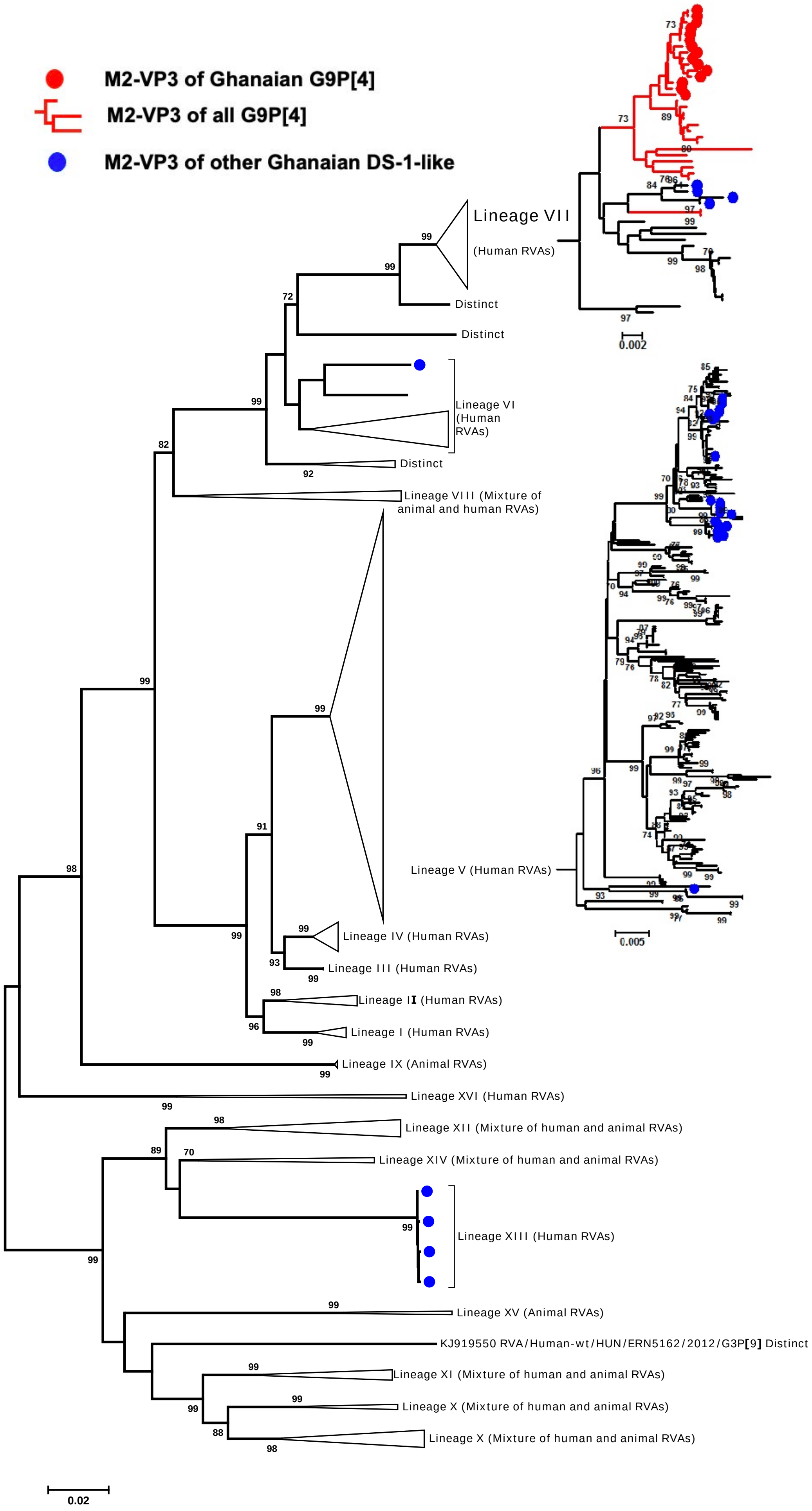

(g) NSP1 tree (Rectangular style): A2 genotype

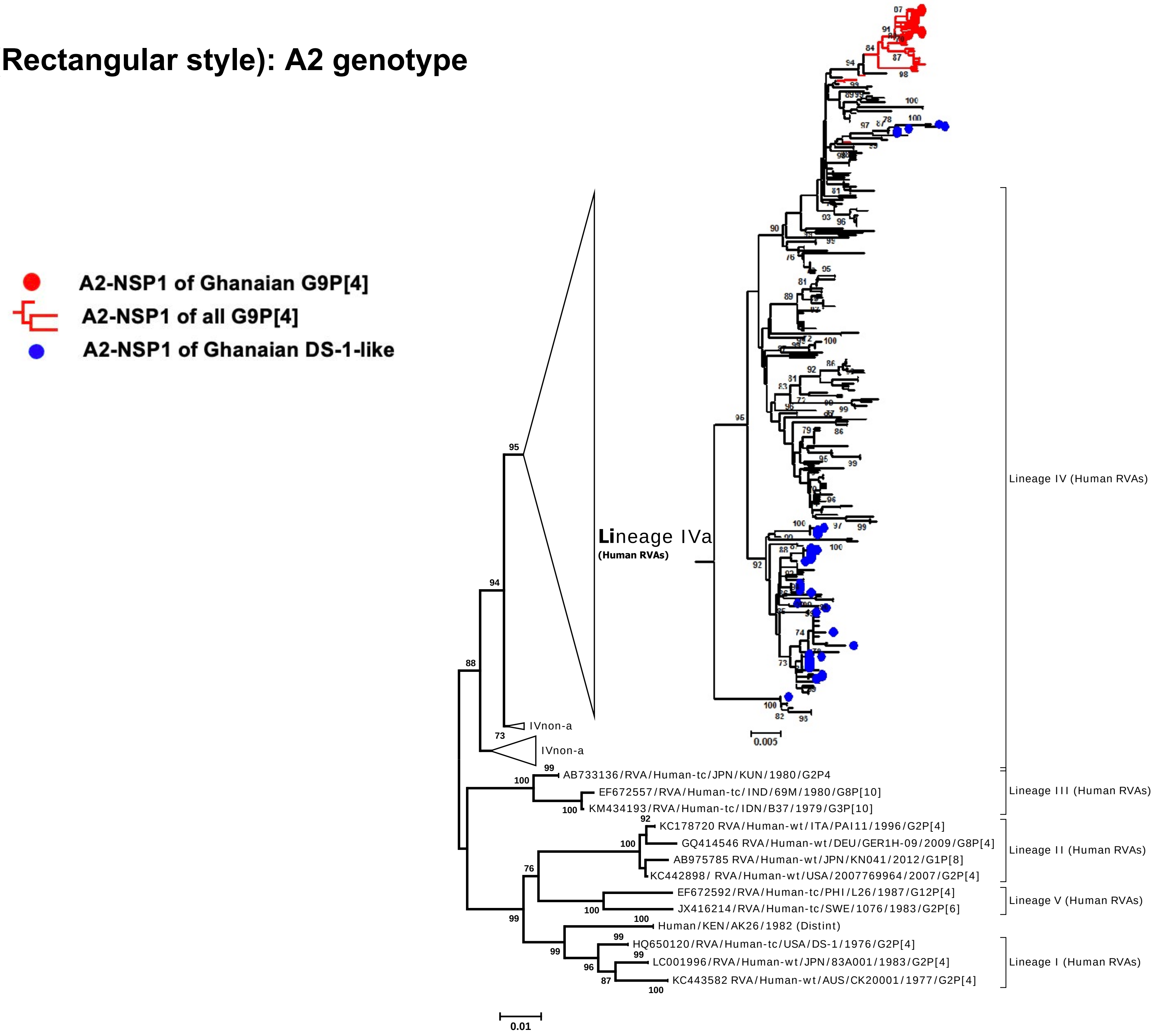

(h) NSP2 tree (Rectangular style): N2 genotype

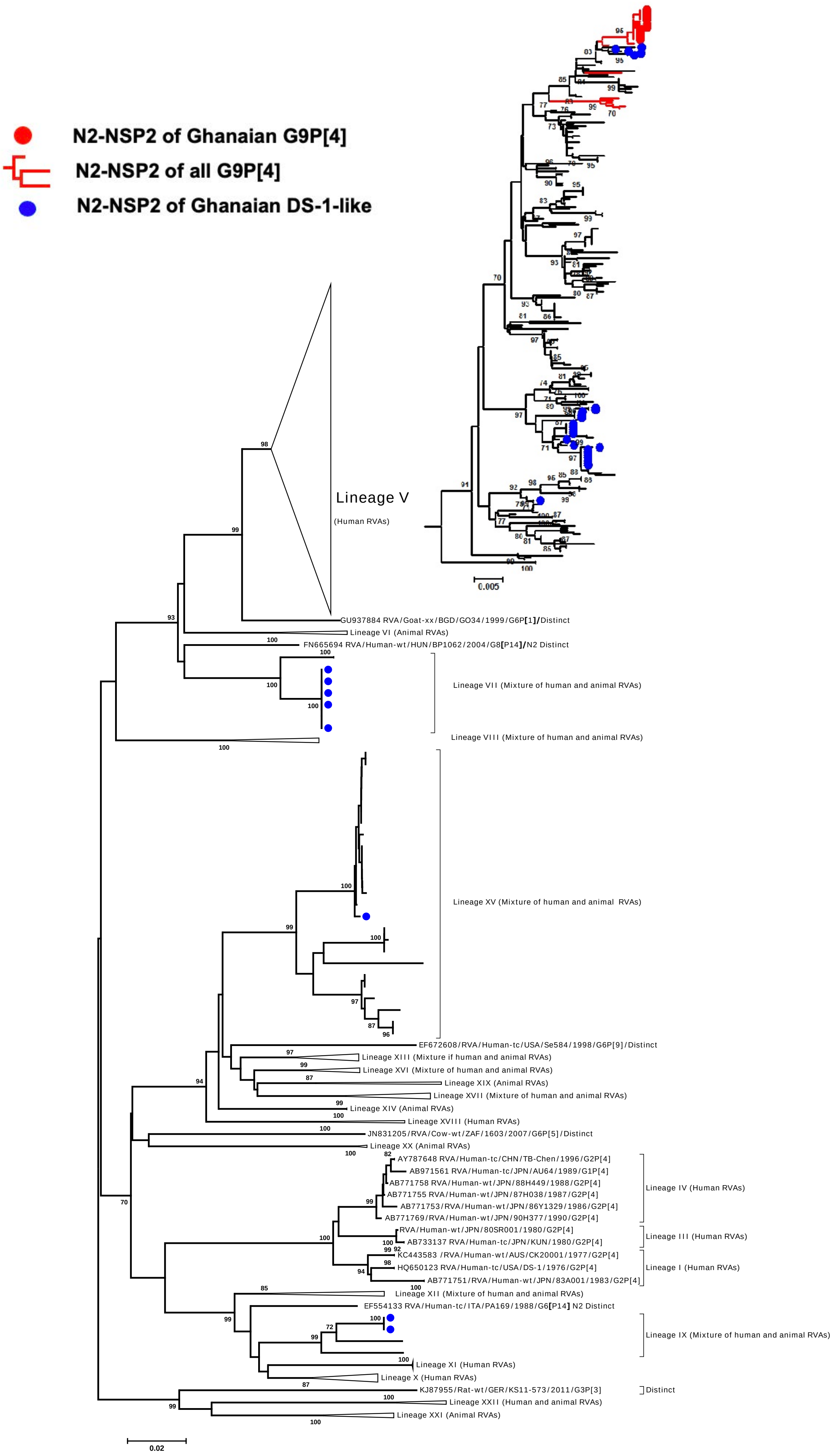

(i) NSP3 tree (Rectangular style): T2 genotype

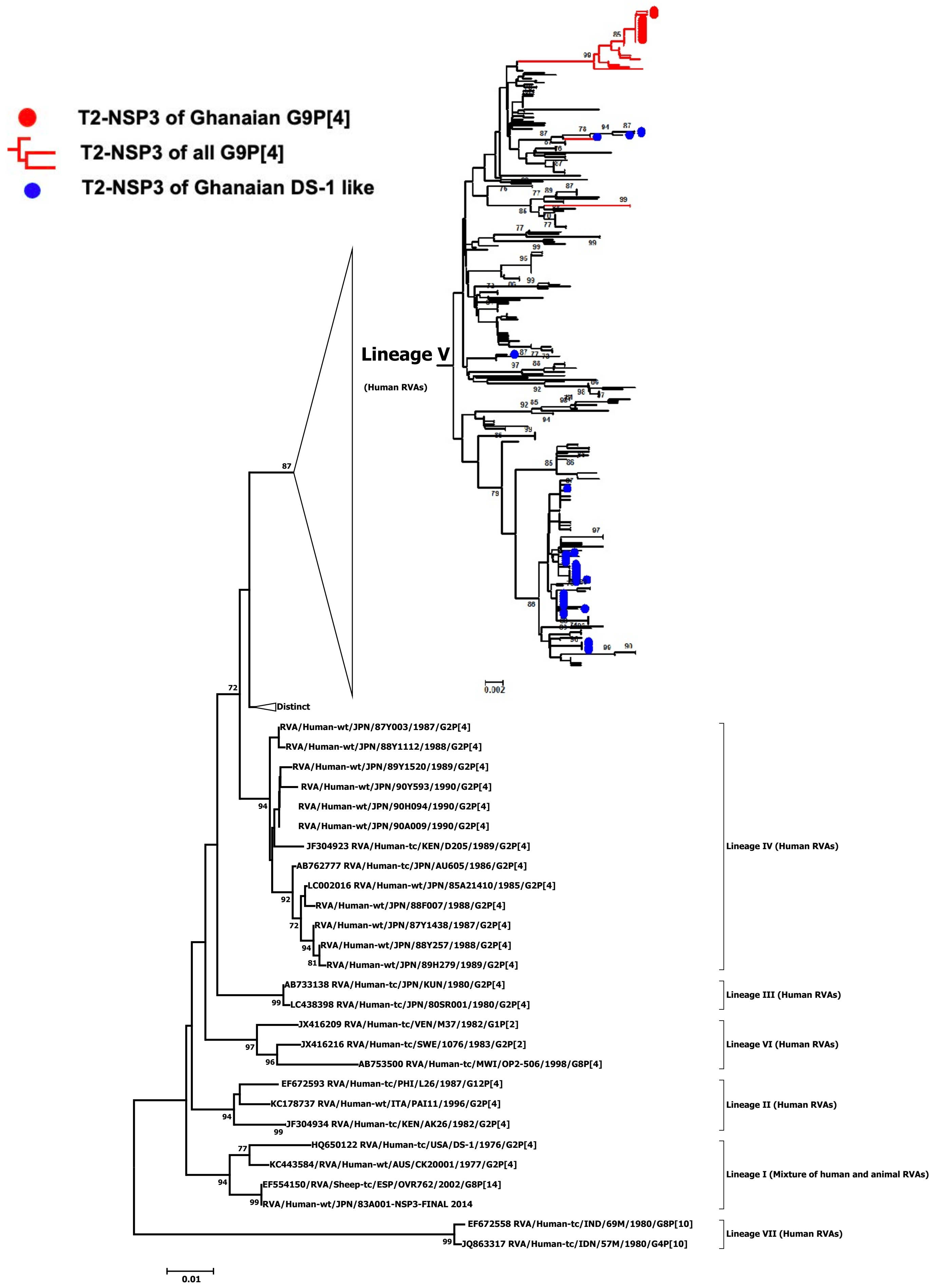

(j) NSP5 tree (Rectangular style): H2 genotype

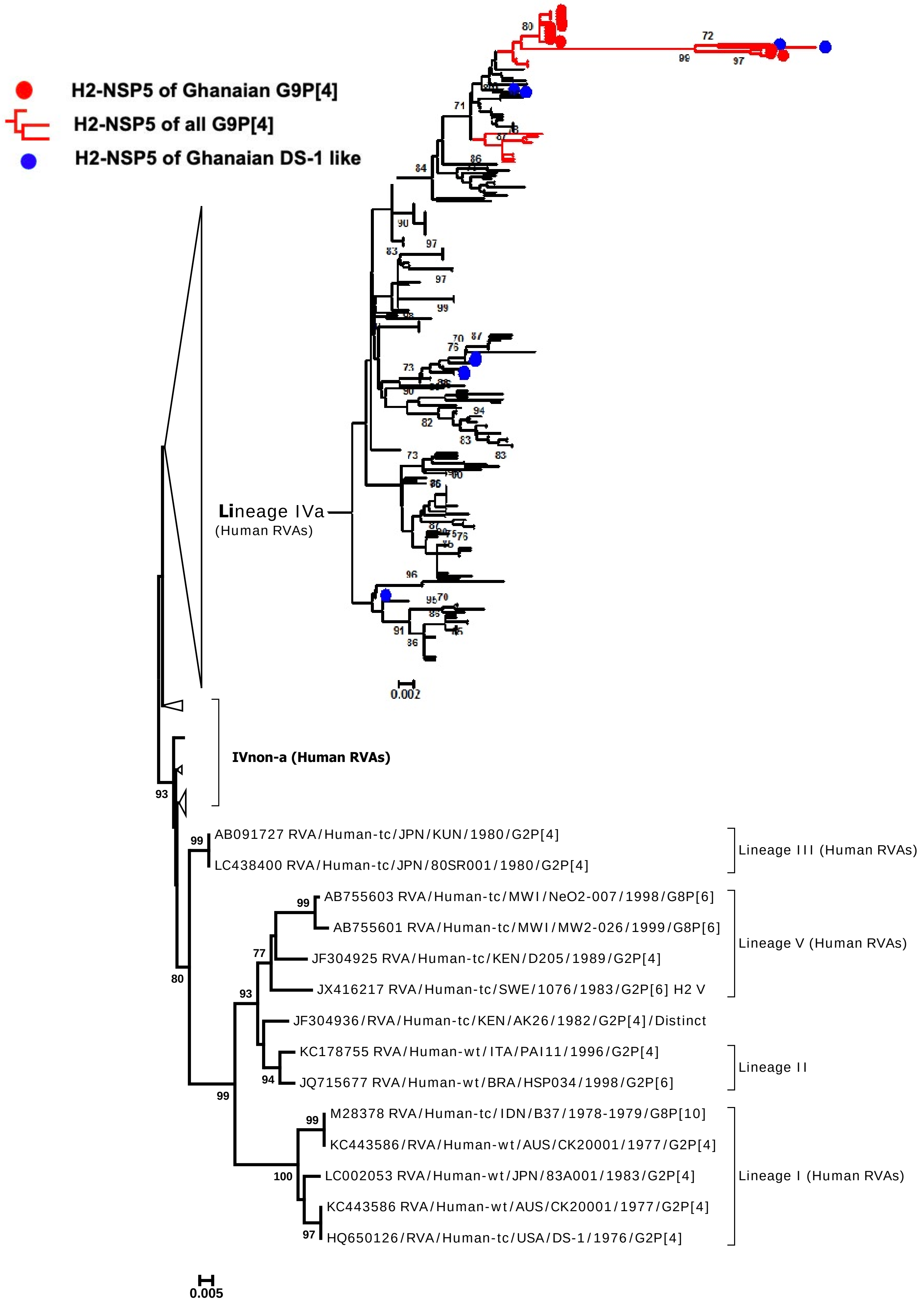

Supplement: Supplementary file 1 [file viruses-15-02453-s001.zip › 04_Figure S3_231218.pdf]
