# Supplementary material for: Emergence of Intergenogroup Reassortant G9P[4] Strains Following Rotavirus Vaccine Introduction in Ghana
Source: Viruses. 2023 Dec 18;15(12):2453. doi: 10.3390/v15122453 (PMC10747750; doi:10.3390/v15122453)
Supplement: Supplementary file 1 [file viruses-15-02453-s001.zip › 05_Table S2-231218.pdf]

Table S2. Distribution of human RVA genotype constellations detected from 1976 to 2006 across 27 countries worldwide

Supplementary 2a. Typical human RVA genotype constellations (n = 735)

	Genogroup	Number of human RVA strains (%)		Genotype constellation: VP7	VP4	VP1	VP2	VP3	NSP1	NSP2	NSP3	NSP4	NSP5	NSP6
1			483 (65.7%)	G1	P[8]	I1	R1	C1	M1	A1	N1	T1	E1	H1
2			6 (0.8%)	G1	P[6]	I1	R1	C1	M1	A1	N1	T1	E1	H1
3			38 (5.2%)	G3	P[8]	I1	R1	C1	M1	A1	N1	T1	E1	H1
4			2 (0.3%)	G3	P[6]	I1	R1	C1	M1	A1	N1	T1	E1	H1
5	Wa	614 (83.8%)	39 (5.3%)	G4	P[8]	I1	R1	C1	M1	A1	N1	T1	E1	H1
6			2 (0.3%)	G4	P[6]	I1	R1	C1	M1	A1	N1	T1	E1	H1
7			12 (1.6%)	G12	P[6]	I1	R1	C1	M1	A1	N1	T1	E1	H1
8			4 (0.5%)	G12	P[8]	I1	R1	C1	M1	A1	N1	T1	E1	H1
9			28 (3.8%)	G9	P[8]	I1	R1	C1	M1	A1	N1	T1	E1	H1
10			75 (10%)	G2	P[4]	I2	R2	C2	M2	A2	N2	T2	E2	H2
11			4 (0.5%)	G2	P[6]	I2	R2	C2	M2	A2	N2	T2	E2	H2
	DS-1	114 (15.3%)												
12			20 (2.7%)	G8	P[4]	I2	R2	C2	M2	A2	N2	T2	E2	H2
13			15 (2.0%)	G8	P[6]	I2	R2	C2	M2	A2	N2	T2	E2	H2
14	AU-1	7 (1%)	7 (1.0%)	G3	P[9]	I3	R3	C3	M3	A3	N3	T3	E3	H3
	Total	735												

Supplementary 2b. Unusual human RVA genotype constellations (n = 119)

58 distinct genotype constellations	Number of human RVAs	Origin of formation	Frequency of appearance	Genotype constellation: VP7	VP4	VP1	VP2	VP3	NSP1	NSP2	NSP3	NSP4	NSP5	NSP6
1	1		Sporadic	G1	P[4]	I2	R2	C2	M2	A2	N2	T2	E2	H2
2	1		Sporadic	G1	P[8]	I1	R1	C1	M1	A1	N1	T2	E1	H1
3	1		Sporadic	G1	P[8]	I1	R1	C1	M1	A2	N1	T1	E1	H1
4	3		Sporadic	G1	P[8]	I1	R1	C1	M2	A1	N1	T2	E1	H1
5	1		Sporadic	G1	P[9]	I1	R1	C1	M2	A1	N2	T2	E1	H1
6	1		Sporadic	G1	P[9]	I1	R1	C1	M3	A1	N1	T2	E1	H1
7	1		Sporadic	G1	P[9]	I1	R3	C3	M3	A1	N1	T3	E3	H3
8	1		Sporadic	G1	P[6]	I1	R1	C1	M1	A1	N1	T2	E1	H1
9	1		Sporadic	G12	P[4]	I2	R2	C2	M1	A2	N1	T2	E2	H1
10	1		Sporadic	G12	P[6]	I1	R1	C1	M1	A1	N1	T2	E1	H1
11	1		Sporadic	G12	P[6]	I2	R2	C2	M2	A2	N1	T2	E6	H2
12	1		Sporadic	G12	P[6]	I2	R2	C2	M2	A2	N2	T2	E1	H2
13	1	Inter-genogoup reassortant	Sporadic	G12	P[6]	I2	R2	C2	M2	A2	N2	T2	E6	H2
14	14		Repeated circulation over a period of 6 years in Japan	G2	P[4]	I2	R2	C2	M2	A2	N1	T2	E2	H2
15	1		Sporadic	G2	P[6]	I2	R2	C2	M2	A2	N1	T2	E2	H2
16	1		Sporadic	G3	P[3]	I1	R1	C1	M1	A1	N1	T1	E1	H1
17	1		Sporadic	G3	P[6]	I2	R2	C2	M2	A2	N2	T2	E2	H2

18	1		Sporadic	G3	P[4]	I2	R2	C2	M2	A2	N2	T2	E2	H2
19	1		Sporadic	G3	P[8]	I1	R1	C1	M1	A1	N2	T1	E1	H1
20	1		Sporadic	G8	P[4]	I2	R2	C2	M2	A2	N2	T2	E2	H2
21	2		Sporadic	G8	P[8]	I2	R2	C2	M2	A2	N2	T2	E2	H2
22	1		Sporadic	G9	P[4]	I1	R1	C1	M1	A1	N1	T1	E1	H1
23	2		Sporadic	G9	P[6]	I2	R2	C2	M2	A2	N1	T2	E2	H2
24	11		Unknow	G9	P[6]	I2	R2	C2	M2	A2	N2	T2	E2	H2
25	1		Sporadic	G9	P[9]	I1	R1	C1	M1	A1	N1	T2	E1	H1
26	1		Sporadic	G1	P[6]	I5	R1	C1	M1	A8	N1	T1	E1	H2
27	1		Sporadic	G10	P[14]	I2	R2	C2	M1	A3	N2	T6	E2	H3
28	2		Sporadic	G11	P[25]	I1	R1	C1	M1	A1	N1	T1	E1	H1
29	1		Sporadic	G12	P[8]	I3	R3	C3	M3	A3	N3	T3	E3	H6
30	3		Sporadic	G12	P[9]	I3	R3	C3	M3	A3	N3	T3	E3	H6
31	1		Sporadic	G3	P[14]	I2	R2	C2	M3	A9	N2	T6	E5	H3
32	1		Sporadic	G3	P[2]	I16	R8	C5	M5	A5	N5	T5	E13	H5
33	1		Sporadic	G3	P[3]	I1	R1	C1	M1	A1	N2	T3	E3	H6
34	1		Sporadic	G3	P[3]	I3	R3	C2	M3	A15	N2	T3	E3	H6
35	1		Sporadic	G3	P[3]	I3	R3	C2	M3	A9	Nx	T3	E3	H6
36	1		Sporadic	G3	P[3]	I3	R3	C2	M3	A9	Nx	T3	E3	Hx
37	2		Sporadic	G3	P[9]	I2	R2	C2	M2	A3	N1	T6	E2	H3
38	1		Sporadic	G3	P[9]	I2	R2	C2	M2	A3	N2	T1	E2	H3
39	1		Sporadic	G3	P[9]	I3	R3	C3	M3	A3	N3	T3	E3	H6
40	1		Sporadic	G4	P[10]	I1	R1	C1	M1	A1	N1	T2	E1	H2
41	1	Interspecies transmission from animal to human	Sporadic	G4	P[6]	I1	R1	C1	M1	A1	N1	T7	E1	H1
42	1		Sporadic	G4	P[6]	I1	R1	C1	M1	A8	N1	T7	E1	H1
43	1		Sporadic	G4	P[6]	I5	R1	C1	M1	A1	N1	T7	E1	H1
44	1		Sporadic	G4	P[6]	I5	R1	C1	M1	A8	N1	T7	E1	H1
45	1		Sporadic	G5	P[6]	I5	R1	C1	M1	A1	N1	T1	E1	H1
46	1		Sporadic	G5	P[8]	I1	R1	C1	M1	A1	N1	T1	E1	H1
47	1		Sporadic	G6	P[1]	I2	R2	C2	M2	A3	N2	T6	E2	H3
48	3		Sporadic	G6	P[14]	I2	R2	C2	M2	A11	N2	T6	E2	H3
49	3		Sporadic	G6	P[14]	I2	R2	C2	M2	A3	N2	T6	E2	H3
50	1		Sporadic	G6	P[6]	I2	R2	C2	M2	A2	N2	T2	E2	H2
51	1		Sporadic	G6	P[9]	I2	R2	C2	M2	A3	N2	T1	E2	H3
52	1		Sporadic	G8	P[1]	I2	R2	C2	M2	A11	N2	T6	E2	H3
53	2		Sporadic	G8	P[1]	I2	R2	C2	M2	A3	N2	T6	E2	H3
54	1		Sporadic	G8	P[10]	I2	R2	C2	M2	A2	N2	T2	E2	H2
55	2		Sporadic	G8	P[14]	I2	R2	C2	M2	A11	N2	T6	E2	H3
56	2		Sporadic	G9	P[19]	I5	R1	C1	M1	A8	N1	T1	E1	H1
57	24	Region-specific	Predominant in neonatal nurseries in India during 2003 and 2004	G10	P[11]	I2	R2	C2	M2	A1	N1	T1	E2	H3
58	1	Unknown the original	Sporadic	G11	P[8]	I1	R1	C1	M1	A1	N1	T1	E1	H1
**Total**	**119**													

Supplementary 2c. Mixed genotype constellations (n = 25)

	Number of human RVAs	Genotype constellation										
		VP7	VP4	VP1	VP2	VP3	NSP1	NSP2	NSP3	NSP4	NSP5	NSP6
1	1	G9	P[9]	I3	R3	C3	M3	A3	N1	T1/T3	E3	H3
2	1	G1	P[4],P[8]	I1	R2	C2	M1	A2	N1/N2	T2	E2	H2
3	1	G1,G10	P[8]	I2	R2	C2	M2	A1	N1	T1	E2	H3
4	1	G12	P[8]	I2	R2	C1,C2	M1	A2	N2	T2	E2	H2
5	4	G1,G3	P[8]	I1	R1	C1	M1	A1	N1	T1	E1	H1
6	1	G2	P[4]	I2	R2,R1	C2	M2	A2	N2	T2	E2	H2
7	1	G2,G9	P[4]	I2	R1,R2, R1	C2	M2	A2	N2	T2	E2	H2
8	1	G1,G9	P[8]	I1,I1	R1	C1,C1	M1,M1	A1,A1	N1,N1	T1,T1	E1,E1	H1,H1
9	3	G2,G9	P[4]	I2	R2	C2	M2	A2	N2	T2	E2	H2
10	1	G4	P[8]	I1	R1	C1	M1	A1	N1	T1	E1,E1	H1
11	1	G1,G4	P[8]	I1	R1	C1	M1	A1	N1	T1	E1	H1
12	1	G1,G4	P[4]	I1/I2	R1/R2	C2	M1	A1	N1/N2	T2	E2	H2
13	1	G1,G9	P[8]	I1	R1	C1	M1	A1	N1	T1	E1	H1
14	1	G12	P[8],P[8]	I3	R3	C3	M3,M1	A3	N1	T3	E3	H6
15	1	G9,G4	P[8]	I1,I1	R1	C1	M1,M1	A1,A1	N1,N1	T1,T1	E1	H1
16	1	G1	P[8]	I1	R1,R1	C1	M1,M1	A1	N1	T1	E1	H1
17	1	G4/G1	P[8]	I1	R1	C1	M1	A1	N1	T1	E1	H1
18	1	G1	P[8]	I1	R1,R1	C1	M1	A1	N1	T1,T1	E1	H1
19	1	G9,G12	P[6]	I1	R1	C1	M1	A1,A1	N1	T1	E1	H1
20	1	G12	P[8]	I1	R1	C1,C1	M1	A1	N1	T1	E1	H1
Total	25											
